# Global burden of ischemic heart disease attributable to ambient and household PM_2.5_ exposure: a comprehensive analysis (1990–2021) from socioeconomics perspective

**DOI:** 10.3389/fpubh.2025.1607163

**Published:** 2025-07-10

**Authors:** Chenran Zhang, Wanghong Su, Huijuan Xi, Shaoru Li, Hongmei Xu, Yue Cheng, Bei Han

**Affiliations:** ^1^School of Public Health, Health Science Center, Xi’an Jiaotong University, Xi'an, China; ^2^Department of Environmental Science and Engineering, Xi'an Jiaotong University, Xi'an, China

**Keywords:** ischemic heart disease, Global Burden of Disease, PM_2.5_, ambient pollution, household air pollution, socio-demographic index

## Abstract

**Objectives:**

Socioeconomic status links to exposure of air pollutants. This study evaluates global PM_2.5_-attributable ischemic heart disease (IHD) burden from 1990 to 2021.

**Methods:**

Using Global Burden of Disease (GBD) 2021 data, PM_2.5_-related IHD burdens were analyzed. Joinpoint regression identified annual percentage changes (AAPCs); Pearson correlation assessed associations with Socio-demographic Index (SDI); Slope Index of Inequality (SII) and Concentration Index (CI) were applied to quantify inequality; Frontier analysis was conducted to evaluate the efficiency of health outcomes relative to development level; Decomposition analysis was performed to identify key drivers of burden changes over time.

**Results:**

From 1990 to 2021, age-standardized rates (ASMR, ASDR) of IHD attributable to ambient PM_2.5_ declined to 20.85 per 100,000 (AAPC = −0.7), with attributable to household PM_2.5_ decreased to 9.02 per 100,000 (AAPC = −2.49). Middle-low SDI regions exhibited the highest increases in ambient PM_2.5_-related burden, whereas high SDI regions showed marked declines (AAPC = −4.31). All regions showed downward in household PM_2.5_-attributable ASMR and ASDR. Disease burden was disproportionately higher among males and older populations. ASMR and ASDR of IHD exhibited a nonlinear association with SDI. PM_2.5_ demonstrated positive correlation in regions with SDI < 0.49, and negative correlation in regions with SDI > 0.623. SII and CI indicated rising inequality in ambient PM_2.5_-related burden. Frontier analysis revealed efficiency gaps in low-SDI regions. Decomposition highlighted population aging and ambient PM_2.5_ exposure as major drivers of burden trends.

**Conclusion:**

Ambient pollution burdens increase in middle-SDI and household pollution impacts focus on low-SDI, which needs prioritizing clean energy and protecting high-risk populations.

## Introduction

Ischemic heart disease (IHD) has been identified as the world’s leading cause of mortality. According to data from the 2021 Global Burden of Disease Study (GBD 2021), IHD was responsible for 9.4 million deaths in 2021, accounting for 16% of the global total, and resulted in 185 million disability-adjusted life years (DALYs) (1). Projections indicate that by 2050, the incidence, prevalence, deaths, and DALYs of global IHD will reach 67.3 million, 510 million, 16 million, and 302 million respectively, representing an increase of 116, 106, 80, and 62% compared with 2021 (2). Therefore, taking effective preventive measures, such as improving lifestyle and controlling metabolic risk factors, are of crucial in reducing the global burden of IHD.

IHD is associated with a number of identifiable and controllable risk factors, including hypertension (3), unhealthy dietary habits (especially high-sodium diets), and high levels of low-density cholesterol (4). Notably, outdoor and indoor solid fuel-derived particulate matter require distinct assessments due to differing sources (e.g., traffic vs. biomass combustion), compositions (e.g., heavy metals vs. organic carbon), and exposure patterns (acute vs. chronic) (5). In addition, recent research has particularly emphasized the impact of air pollution, especially fine particulate matter (PM_2.5_), on IHD. It was reported that a variety of chemical components (PAHs, heavy metals, etc.) in PM_2.5_ ultimately increase the risk of IHD by triggering a local inflammatory response and releasing large amounts of pro-inflammatory cytokines, leading to endothelial dysfunction (6–9). A large number of researches had substantiated the existence of a robust relationship between PM_2.5_ exposure and IHD from diverse vantage points, and that this relationship is universal, stable and specific.

According to the 2021 GBD statistical estimates, the contribution rate of particulate pollutants to global IHD deaths was 27.73%. Among these pollutants, atmospheric PM_2.5_ contributed to 19.23% of global IHD deaths, which is 2.27 times the proportion caused by household PM_2.5_ pollution from solid fuel use (8.49%). The epidemiological profile of PM_2.5_-associated IHD burden remains inadequately characterized at a global scale, particularly regarding temporal trends, regional heterogeneity, and demographic impacts. Leveraging updated data from the GBD, this investigation systematically evaluates the evolving disease burden of PM_2.5_-attributable IHD from 1990 to 2021 across global and regional dimensions, employing mortality and DALYs as key metrics. The research findings aim to inform evidence-based public health strategies and healthcare policy formulation.

## Methods

### Data sources

The 2021 GBD database, curated by the Institute for Health Metrics and Evaluation (IHME), offers comprehensive epidemiological analyses of 371 diseases and injuries alongside 87 attributable risk factors across 204 countries and territories, 5 Socio-demographic Index (SDI) quintiles, and 21 geographic regions. The GBD database applies methods to address missing data and adjust for confounding factors. All datasets, analytical outputs, and methodological details are publicly accessible through the GBD 2021 platform,^1^ enabling unrestricted access to granular data, statistical models, and protocol documentation (10, 11).

Ischemic heart disease (IHD) is coded within the range I20-I25 in the International Classification of Diseases, 10th Revision (ICD-10). The specific classifications are as of I20 (Angina Pectoris), I21 (Acute Myocardial Infarction), I22 (Subsequent Myocardial Infarction), I24 (Other Acute Ischemic Heart Diseases), and I25 (Chronic Ischemic Heart Disease). These codes cover various types of ischemic heart disease, ranging from acute episodes to chronic conditions.

### Estimation of PM_2.5_ exposure

In the GBD study, PM_2.5_ encompasses both ambient particulate matter pollution and household air pollution from solid fuels. Ambient particulate matter pollution is defined as the population-weighted annual average mass concentration of outdoor PM_2.5_ exposure, derived through integration of satellite observations of atmospheric aerosols, ground-level measurements, chemical transport model simulations, population estimates, and land-use data (10, 11). Household air pollution (HAP) exposure from solid fuels is estimated based on both the proportion of individuals using solid cooking fuels and the corresponding PM_2.5_ exposure levels (1, 11). In GBD 2021, solid fuels include coal, wood, charcoal, crop residues, dung, and agricultural waste (11). Data on household air pollution are sourced from Demographic and Health Surveys (DHS)^2^ and Living Standards Measurement Surveys (LSMS)^3^ (2).

### Statistical analysis

Mortality counts, DALYs, ASMRs, ASDRs were used as metrics to quantify the PM_2.5_-attributable ischemic heart disease burden. These data were extracted from the GBD Results Tool.

This study employed Joinpoint software (Version 4.9.1.0) to calculate the APCs and AAPCs (12), along with their 95% confidence intervals (95% CIs). APCs were used to identify specific segments of linear trends over the study period, while AAPCs provided an estimate of the overall change across the entire 32-year span from 1990 to 2021, thereby analyzing the magnitude and direction of trends in IHD mortality and DALYs. The Joinpoint software utilized a grid search method with six Joinpoints and Monte Carlo permutation tests to analyze mortality data and optimize the model. The Joinpoint regression model is expressed as follows:


$$\ln(\text{ASDR or ASMR})=\alpha +\beta_{i}x+\varepsilon$$

$$\text{APCs}=100\times \{\exp(\beta_{i})-1\}$$

AAPCs={exp(∑ωiβi∑ωi)−1}

In the specified model, x represents the calendar year. The parameter $\beta_{i}$ denotes the slope coefficient for each segment within the partitioned time intervals, while $\omega_{i}$ corresponds to the duration of each segment. Trends in ASMRs or ASDRs were classified as follows: an upward trend was identified if the lower bound of the 95% CI for the AAPC > 0; a downward trend was determined if the upper bound of the 95% CI < 0. Otherwise, ASMRs or ASDRs were considered stable (13).

Pearson correlation coefficients were employed to assess the associations between SDI and ASMRs or ASDRs. The expected relationships between SDI and ASMR/ASDR were derived using locally estimated scatterplot smoothing (LOESS) fitted to data spanning 1990–2021. Stochastic frontier analysis (SFA) modeled the relationship between sociodemographic progress (SDI) and achievable IHD burden reduction. Age-standardized rates (ASMR/ASDR) were regressed against SDI to estimate region-specific efficiency scores, quantifying the gap between observed burden and the theoretical minimum (efficient frontier) at each SDI level (14). Absolute Slope Index of Inequality (SII) and relative Concentration Index (CI) inequalities in CVD burden were assessed across SDI-ranked countries. SII quantifies the absolute burden gap between highest/lowest SDI; negative values indicate concentration in disadvantaged populations. CI measures relative inequality via Lorenz deviation: negative values denote disproportionate burden in low-SDI countries. Both indices assume zero means perfect equality. Analyses used national-level age-standardized burden metrics (15). Additionally, we used a recently developed decomposition method to attribute changes in PM_2.5_ associated total IHD ASMR and ASDR to population growth, population aging, and ASMR or ASDR changes from 1990 to 2021 in 21 GBD regions and five SDI groups (14, 16). This study used the Bayesian age-period-cohort (BAPC) model with INLA to predict IHD burden globally from 2022 to 2046, leveraging GBD population estimates (1990–2046). The BAPC model demonstrated superior accuracy (*p* < 0.05) (17). All analyses were implemented in R software (version 4.4.1).^4^

## Results

### IHD burden attributable to ambient PM_2.5_ pollution from 1990 to 2021

#### Global burden of IHD attributable to ambient PM_2.5_

Globally, the ASMR of IHD attributable to ambient PM_2.5_ decreased from 25.95 (95% UI: 17.27~34.24) per 100,000 population in 1990 to 20.85 (95% UI: 14.63~27.57) per 100,000 in 2021. The AAPC was −0.7 (95% CI: −1.05~−0.36) (Supplementary Figure S1a) globally, with males showing a slightly smaller magnitude of decline (AAPC: -0.44; 95% CI: −0.81~−0.06) compared to the global average (Supplementary Figure S1b), while females exhibited a greater reduction (AAPC: −1.04; 95% CI: −1.35~−0.73) (Supplementary Figure S1c). Similarly, the ASDR associated with ambient PM_2.5_ declined from 479.87 (95% UI: 328.35~640.66) per 100,000 in 1990~427.81 (95% UI: 299.61~564.17) per 100,000 in 2021. The global average AAPC for ASDR was −0.41 (95% CI: −0.79~−0.04) (Table 1; Supplementary Figure S1d), with males demonstrating a less pronounced decline (AAPC: −0.27; 95% CI: −0.67~−0.13) relative to the global trend (Supplementary Figure S1e), whereas females experienced a more substantial reduction (AAPC: −0.66; 95% CI: −0.98~−0.33) (Table 1; Supplementary Figure S1f). The temporal patterns of DALYs closely paralleled those of mortality trends (Table 1).

**Table 1:** ASMR, ASDR, and AAPCs attributed to ambient PM_2.5_ in 1990 and 2021.

Location	Gender	ASMR (95%UI)			ASDR (95%UI)		
		1990	2021	1990–2021 AAPC	1990	2021	1990–2021 AAPC
Global	Both	25.55 (17.27, 34.24)	20.85 (14.63, 27.57)	−0.7^*^ (−1.05, −0.36)	479.87 (328.35, 640.66)	427.81 (299.61, 564.17)	−0.41^*^ (−0.79, −0.04)
	Male	30.54 (21, 40.43)	27.23 (19.23, 35.33)	−0.44^*^ (−0.81, −0.06)	616.26 (424.53, 819.07)	576.62 (404.84, 747.45)	−0.27 (−0.67, 0.13)
	Female	21.26 (14.25, 28.99)	15.63 (10.72, 20.85)	−1.04^*^ (−1.35, −0.73)	354.54 (239.93, 475.6)	293.72 (197.79, 391.6)	−0.66^*^ (−0.98, −0.33)
Low	Both	8.77 (5.33, 12.75)	11.39 (7.19, 16.76)	0.89^*^ (0.44, 1.34)	193.76 (116.93, 284.59)	240.42 (150.86, 354.45)	0.75^*^ (0.3, 1.19)
	Male	10.81 (6.47, 16.31)	15.42 (9.56, 22.51)	1^*^ (0.53, 1.47)	246.47 (147.73, 374.21)	329.89 (205.14, 482.83)	0.8^*^ (0.35, 1.24)
	Female	6.73 (4.06, 9.99)	7.75 (4.67, 11.98)	0.48^*^ (0.03, 0.94)	139.54 (84.01, 210.42)	155.99 (94.06, 241.8)	0.4 (−0.08, 0.88)
Low-middle	Both	14.12 (9.19, 19.68)	25.35 (15.34, 35.75)	1.94^*^ (1.47, 2.41)	311.08 (200.62, 434.17)	563.56 (344.13, 790.84)	1.91^*^ (1.64, 2.18)
	Male	16.77 (10.66, 23.7)	33.27 (20.32, 46.35)	2.21^*^ (1.86, 2.56)	387.99 (246.92, 547.95)	758.1 (461.05, 1062.25)	2.17^*^ (1.86, 2.49)
	Female	11.43 (7.12, 16.35)	18.39 (10.75, 27.1)	1.6^*^ (1.31, 1.9)	231.4 (144.92, 331.09)	383.01 (225.62, 566.49)	1.65^*^ (1.38, 1.92)
Middle	Both	17.61 (11.05, 24.7)	29.48 (19.55, 39.08)	1.62^*^ (1.38, 1.85)	353.08 (222.21, 489.28)	580.44 (381.77, 764.95)	1.58^*^ (1.39, 1.77)
	Male	22.36 (14.17, 30.88)	39.02 (26.4, 50.87)	1.73^*^ (1.46, 2.01)	462.55 (294.94, 642.77)	782.9 (534.6, 1016.15)	1.66^*^ (1.45, 1.87)
	Female	13.59 (8.41, 19)	21.81 (13.35, 29.42)	1.52^*^ (1.3, 1.73)	250.59 (153.85, 351.33)	400.54 (245.38, 533.76)	1.48^*^ (1.33, 1.64)
High-middle	Both	40.02 (25.87, 55.56)	25.78 (17.84, 33.84)	−1.47^*^ (−1.9, −1.03)	725.45 (471.88, 1005.48)	466.53 (328.68, 609.05)	−1.46^*^ (−2.07, −0.85)
	Male	47.17 (31.23, 64.29)	32.82 (22.94, 42.65)	−1.23^*^ (−1.78, −0.67)	932.13 (616.89, 1277.94)	620.59 (436.44, 809.76)	−1.35^*^ (−1.99, −0.72)
	Female	34.25 (21.72, 48.06)	20.51 (13.62, 27.3)	−1.62^*^ (−2.02, −1.21)	545.37 (350.67, 764.6)	334.74 (226.34, 441.21)	−1.63^*^ (−2.06, −1.19)
High	Both	26.12 (15.9, 36.69)	6.71 (4.54, 9)	−4.31^*^ (−4.5, −4.11)	489.54 (299.11, 688.83)	140.07 (96.36, 185.43)	−3.98^*^ (−4.21, −3.74)
	Male	35.1 (21.28, 49.44)	9.04 (6.09, 12.21)	−4.29^*^ (−4.47, −4.12)	697.38 (425.34, 984.95)	199.15 (136, 264.28)	−3.99^*^ (−4.19, −3.78)
	Female	19.65 (11.85, 27.79)	4.71 (3.04, 6.35)	−4.52^*^ (−4.77, −4.28)	321.16 (196.26, 449.29)	84.85 (57.36, 112.64)	−4.23^*^ (−4.37, −4.09)
GBD regions							
Andean Latin America	Both	22.3 (10.69, 35.26)	12.1 (7.03, 18.15)	−2.16^*^ (−2.43, −1.93)	440.54 (212.75, 692.51)	236.56 (140.68, 349.94)	−2.17^*^ (−2.44, −1.88)
	Male	27.09 (13.44, 42.07)	14.82 (9.02, 21.39)	−2.04^*^ (−2.22, −1.88)	564.74 (278.95, 871.73)	307.91 (189.1, 447.88)	−2.04^*^ (−2.26, −1.82)
	Female	17.84 (8.06, 29.12)	9.65 (5.15, 14.66)	−2.24^*^ (−2.48, −2.02)	322.68 (146.89, 527.6)	170.44 (89.8, 262.83)	−2.09^*^ (−2.29, −1.89)
Australasia	Both	9.48 (0.33, 25.92)	3.35 (1.84, 5.09)	−3.26^*^ (−3.7, −2.7)	173.2 (6.01, 472.44)	58.13 (31.57, 87.98)	−3.31^*^ (−3.74, −2.78)
	Male	12.58 (0.42, 34.31)	4.61 (2.5, 7.02)	−3.08^*^ (−3.52, −2.54)	242.69 (8.11, 660.19)	85.7 (46.66, 129.55)	−3.09^*^ (−3.52, −2.57)
	Female	7.08 (0.26, 19.41)	2.27 (1.22, 3.47)	−3.64^*^ (−4.08, −3.09)	113.26 (4.12, 310.57)	33.18 (17.89, 50.09)	−3.84^*^ (−4.27, −3.3)
Caribbean	Both	24.28 (8.28, 45.09)	16.61 (8.38, 27.54)	−1.11^*^ (−1.21, −0.99)	461.26 (155.91, 859.45)	340.63 (169.69, 571.82)	−0.8^*^ (−0.87, −0.71)
	Male	28.65 (9.94, 53.7)	20.37 (10.45, 34.16)	−0.98^*^ (−1.1, −0.85)	568.6 (198.39, 1069.24)	442.56 (220.56, 741.71)	−0.63^*^ (−0.72, −0.53)
	Female	20.29 (6.43, 38.19)	13.27 (6.38, 21.56)	−1.31^*^ (−1.42, −1.22)	360.85 (114.71, 681.95)	247.48 (119.79, 409.05)	−1.04^*^ (−1.12, −0.94)
Central Asia	Both	47.44 (17.96, 88.22)	58.77 (38.36, 79.22)	0.64^*^ (0.45, 0.92)	922.86 (356.2, 1709.3)	1078.17 (699.69, 1447.15)	0.44^*^ (0.26, 0.7)
	Male	65.23 (25.38, 119.39)	76.55 (51, 102.76)	0.37^*^ (0.19, 0.62)	1329.67 (527.13, 2445.88)	1452.7 (969.63, 1934.6)	0.21^*^ (0.03, 0.47)
	Female	36.6 (12.85, 69.05)	47.04 (29.7, 63.37)	0.7^*^ (0.51, 0.97)	633.82 (224.19, 1200.94)	795.04 (498.39, 1077.19)	0.68^*^ (0.51, 0.91)
Central Europe	Both	60.06 (29, 91.37)	23.53 (16.34, 30.91)	−3.05^*^ (−3.12, −2.98)	1148.48 (562.51, 1737.27)	415.66 (295.9, 544.59)	−3.32^*^ (−3.39, −3.25)
	Male	81.42 (40.96, 121.97)	30.31 (21.28, 39.85)	−3.2^*^ (−3.26, −3.14)	1661.41 (835.23, 2480.1)	578.36 (414.02, 756.18)	−3.44^*^ (−3.51, −3.36)
	Female	44.89 (20.67, 69.45)	18.37 (12.33, 24.05)	−2.91^*^ (−2.97, −2.83)	738.61 (335.28, 1140.79)	279.5 (187.63, 365.39)	−3.17^*^ (−3.25, −3.09)
Central Latin America	Both	25.92 (13.51, 39.48)	14.07 (8.92, 19.44)	−1.92^*^ (−1.99, −1.85)	500.09 (260.5, 763.15)	271.36 (174.17, 377.03)	−1.93^*^ (−2.01, −1.84)
	Male	29.48 (15.72, 44.23)	17.75 (11.29, 24.91)	−1.6^*^ (−1.7, −1.5)	614.81 (330.44, 930.39)	365.92 (233.91, 511.19)	−1.63^*^ (−1.73, −1.51)
	Female	22.57 (11.78, 34.66)	10.97 (6.52, 15.23)	−2.27^*^ (−2.36, −2.18)	391.99 (205.35, 605.04)	189.42 (116.32, 265.53)	−2.29^*^ (−2.38, −2.21)
Central Sub-Saharan Africa	Both	7.7 (3.85, 12.77)	9.62 (5.3, 15.22)	0.76^*^ (0.69, 0.82)	158.74 (79.45, 265.51)	197.1 (108.04, 314.49)	0.73^*^ (0.66, 0.8)
	Male	10.15 (5.11, 17.32)	13.36 (7.37, 20.66)	0.92^*^ (0.86, 0.98)	223.54 (110.26, 382.32)	280.6 (150.84, 438.9)	0.76^*^ (0.7, 0.84)
	Female	5.55 (2.7, 9.06)	6.91 (3.59, 11.31)	0.74^*^ (0.66, 0.83)	101.6 (48.61, 165.46)	129.53 (66.7, 211.44)	0.8^*^ (0.73, 0.89)
East Asia	Both	9.09 (3.92, 15.56)	31.19 (19.3, 41.55)	4.04^*^ (3.91, 4.19)	168.74 (73.31, 289.16)	519.47 (325.37, 695.98)	3.67^*^ (3.55, 3.8)
	Male	12.35 (5.32, 21.19)	42.22 (26.92, 57.02)	3.98^*^ (3.82, 4.15)	224.6 (96.48, 389.35)	703.25 (447.13, 963.92)	3.69^*^ (3.56, 3.84)
	Female	7 (2.96, 12.77)	23.7 (13.67, 32.98)	4.02^*^ (3.93, 4.11)	123.73 (54.52, 229.22)	369 (210.23, 512.03)	3.59^*^ (3.51, 3.67)
Eastern Europe	Both	77.21 (39.91, 117.04)	30.78 (18.6, 45.9)	−3.03^*^ (−3.29, −2.73)	1415.39 (730.58, 2145.12)	566.47 (347.77, 847.07)	−2.81^*^ (−3.08, −2.61)
	Male	104.96 (53.88, 158.36)	40.37 (24.65, 59.6)	−3.08^*^ (−3.28, −2.87)	2079.36 (1071.67, 3143.43)	813.29 (493.41, 1204.09)	−3.03^*^ (−3.24, −2.79)
	Female	62.9 (32.61, 95.66)	24.61 (14.65, 37.18)	−3.15^*^ (−3.41, −2.87)	996.46 (518.02, 1509.64)	391.03 (233.85, 593.49)	−2.92^*^ (−3.27, −2.66)
Eastern Sub-Saharan Africa	Both	2.49 (1.52, 3.84)	3.54 (2.04, 5.64)	1.26^*^ (1.14, 1.37)	55.97 (34.56, 84.79)	75.7 (44.05, 118.82)	1.07^*^ (0.96, 1.17)
	Male	3.23 (1.92, 5.08)	5.02 (2.96, 7.87)	1.54^*^ (1.43, 1.64)	75.66 (45.38, 118.33)	110.26 (64.92, 171.01)	1.31^*^ (1.2, 1.41)
	Female	1.77 (1.08, 2.67)	2.3 (1.26, 3.78)	0.95^*^ (0.83, 1.06)	36.51 (22.05, 55.49)	44.82 (24.62, 73.15)	0.77^*^ (0.65, 0.87)
High-income Asia Pacific	Both	9.19 (2.43, 18.09)	3.72 (2.13, 5.53)	−2.9^*^ (−2.98, −2.8)	164.26 (44.66, 315.77)	73.02 (42.08, 107.31)	−2.63^*^ (−2.69, −2.56)
	Male	11.75 (3.03, 23.19)	5.19 (2.93, 7.67)	−2.62^*^ (−2.7, −2.54)	224.59 (60.26, 437.9)	109.18 (62.76, 159.93)	−2.33^*^ (−2.39, −2.26)
	Female	7.33 (1.85, 14.43)	2.46 (1.38, 3.71)	−3.54^*^ (−3.63, −3.45)	114.88 (31.24, 224.17)	39.28 (22.51, 58.06)	−3.51^*^ (−3.59, −3.43)
High-income North America	Both	23.22 (8.82, 41.45)	3.53 (1.64, 5.85)	−5.76^*^ (−6.01, −5.52)	437.99 (165.72, 786.19)	68.38 (31.23, 113.23)	−5.69^*^ (−6.01, −5.43)
	Male	31.29 (11.71, 56.26)	4.81 (2.2, 7.98)	−5.75^*^ (−6.02, −5.5)	621.87 (232.35, 1114.48)	96.88 (44.31, 160.79)	−5.71^*^ (−6, −5.46)
	Female	17.36 (6.64, 30.85)	2.48 (1.16, 4.17)	−5.94^*^ (−6.16, −5.69)	290.07 (111.66, 515.6)	43.32 (20.02, 71.72)	−5.83^*^ (−6.06, −5.6)
North Africa and Middle East	Both	58.49 (39.64, 75.97)	56.21 (42.41, 70.12)	−0.22^*^ (−0.32, −0.09)	1218.59 (826.94, 1581.55)	1129.04 (856.31, 1402.57)	−0.38^*^ (−0.47, −0.26)
	Male	66.6 (46.48, 86.82)	62.92 (47.53, 78.25)	−0.26^*^ (−0.35, −0.16)	1474.1 (1029.91, 1907.82)	1345.8 (1021.55, 1668.81)	−0.44^*^ (−0.52, −0.37)
	Female	50.02 (33.27, 65.68)	49.16 (37.04, 62.42)	−0.07 (−0.19, 0.03)	950.42 (628.37, 1242.55)	898.2 (679.25, 1138.57)	−0.23^*^ (−0.35, −0.11)
Oceania	Both	7.61 (2.34, 18.14)	10.28 (3.73, 21.63)	0.96^*^ (0.91, 1)	178.36 (52.64, 438.11)	235.78 (83.84, 500.93)	0.9^*^ (0.86, 0.95)
	Male	10.08 (3.09, 23.9)	13.31 (4.9, 27.9)	0.89^*^ (0.83, 0.94)	247.8 (73.96, 602.68)	321.01 (114, 679.01)	0.84^*^ (0.78, 0.89)
	Female	5.07 (1.5, 12.25)	7.12 (2.48, 14.72)	1.09^*^ (1.05, 1.13)	104.3 (30.7, 255.35)	145.3 (50.45, 312.86)	1.07^*^ (1.03, 1.11)
South Asia	Both	12.03 (6.55, 19.16)	31.37 (18.8, 43.77)	3.04^*^ (2.88, 3.17)	288.24 (156.91, 462.21)	703.17 (420.75, 977.58)	2.84^*^ (2.66, 2.97)
	Male	15.54 (8.02, 24.94)	42.07 (25.71, 58.72)	3.12^*^ (2.96, 3.25)	381.67 (199.67, 612.06)	953.09 (582, 1347.24)	2.88^*^ (2.71, 3.01)
	Female	8.21 (4.19, 13.6)	21.73 (11.76, 31.65)	3.17^*^ (2.98, 3.32)	185.26 (95.95, 306.15)	464.13 (252.02, 672.73)	2.9^*^ (2.72, 3.05)
Southeast Asia	Both	11.46 (5.08, 19.86)	17.82 (10.58, 26.08)	1.4^*^ (1.3, 1.51)	255.86 (112.89, 445.64)	385.73 (230.05, 558.15)	1.29^*^ (1.18, 1.42)
	Male	14.67 (6.49, 25.94)	23 (13.79, 32.46)	1.42^*^ (1.32, 1.55)	340.59 (151.07, 597.44)	522.57 (315.54, 739.4)	1.37^*^ (1.27, 1.48)
	Female	8.8 (3.81, 15.85)	13.47 (7.3, 20.14)	1.35^*^ (1.25, 1.46)	180.52 (77.92, 323.35)	263.3 (144.36, 399.9)	1.18^*^ (1.1, 1.25)
Southern Latin America	Both	24.18 (10.53, 40.76)	8.17 (4.72, 12.67)	−3.34^*^ (−3.46, −3.23)	457.46 (201.42, 770.37)	161.61 (92.1, 245.7)	−3.22^*^ (−3.35, −3.11)
	Male	32.18 (14.15, 53.75)	11.14 (6.38, 16.96)	−3.31^*^ (−3.44, −3.2)	657.51 (291.39, 1099.42)	236.26 (133.97, 355.66)	−3.2^*^ (−3.31, −3.09)
	Female	17.83 (7.46, 30.64)	5.81 (3.34, 9.14)	−3.43^*^ (−3.59, −3.29)	289.54 (120.53, 495.96)	98.45 (56.04, 153.07)	−3.31^*^ (−3.44, −3.2)
Southern Sub-Saharan Africa	Both	12.06 (7.14, 17.09)	13.71 (8.99, 18.55)	0.42^*^ (0.27, 0.56)	263.15 (158.16, 370.95)	276.19 (180.79, 372.47)	0.11 (−0.03, 0.25)
	Male	16.63 (10.52, 23.18)	17.63 (11.7, 24.05)	0.2^*^ (0.06, 0.29)	381.23 (241.22, 522.95)	379.69 (251.54, 513.16)	−0.08 (−0.19, 0.04)
	Female	8.73 (4.97, 12.79)	10.97 (7.02, 15.17)	0.81^*^ (0.6, 1.01)	168.93 (97.24, 249.26)	197.79 (127.19, 269.94)	0.71^*^ (0.5, 0.9)
Tropical Latin America	Both	16.12 (5.32, 30.96)	7.12 (3.97, 10.86)	−2.57^*^ (−2.64, −2.51)	348.99 (116.58, 664.54)	162.65 (91.76, 247.72)	−2.49^*^ (−2.56, −2.41)
	Male	20.15 (6.74, 38.54)	9.38 (5.25, 14.36)	−2.36^*^ (−2.42, −2.29)	465.46 (156.56, 888.02)	223.48 (127.58, 342.43)	−2.36^*^ (−2.42, −2.29)
	Female	12.56 (3.84, 23.83)	5.29 (2.91, 8.15)	−2.69^*^ (−2.78, −2.58)	243.15 (74.33, 463.54)	110.6 (61.17, 169.82)	−2.4^*^ (−2.51, −2.3)
Western Europe	Both	28.76 (14.29, 45.68)	4.5 (2.89, 6.25)	−5.89^*^ (−5.98, −5.79)	530.48 (263.9, 837.53)	79.79 (51.46, 110.17)	−6.03^*^ (−6.13, −5.95)
	Male	39.4 (19.61, 62.11)	6.35 (4.12, 8.8)	−5.81^*^ (−5.9, −5.72)	774.23 (385.74, 1,216)	119.12 (76.67, 164.31)	−5.95^*^ (−6.05, −5.86)
	Female	21.26 (10.53, 34.14)	3.03 (1.87, 4.3)	−6.15^*^ (−6.26, −6.04)	336.71 (167.08, 538.59)	45.41 (28.68, 63.9)	−6.35^*^ (−6.44, −6.25)
Western Sub-Saharan Africa	Both	11.64 (6.41, 18.16)	14.84 (7.96, 24.16)	0.9^*^ (0.77, 1.01)	231.26 (128.94, 353.41)	279.93 (150.73, 460.31)	0.67^*^ (0.55, 0.79)
	Male	13.19 (7.21, 20.77)	18.11 (9.64, 29.35)	1.07^*^ (0.94, 1.2)	270.81 (148.23, 422.9)	352.07 (189.39, 576.76)	0.9^*^ (0.79, 1.01)
	Female	10.14 (5.35, 16.94)	12 (6.22, 20.22)	0.66^*^ (0.53, 0.77)	189.64 (100.35, 310.79)	215.98 (108.98, 365.49)	0.53^*^ (0.4, 0.64)

^*^Indicate that the AAPC is significantly different from zero at α = 0.05 level.

#### Global burden of IHD among different gender and age

The global burden of IHD attributable to ambient PM_2.5_ pollution exhibited significant gender disparities, with males bearing a disproportionately higher burden. In 2021, the ASMR for males was 27.23 (95% UI: 19.23~35.33) per 100,000 population, nearly double the female ASMR of 15.63 (95% UI: 10.72~20.85) (Supplementary Figure S1a). Similarly, the ASDR for males reached 576.62 (95% UI: 404.84~747.45) per 100,000, substantially exceeding the female ASDR of 293.72 (95% UI: 197.79~391.60) (Table 1; Supplementary Figure S1b). Over the continuous 31-year observation period, both genders demonstrated similar trajectories in ASMR and ASDR reductions; however, females experienced a more pronounced decline compared to males (Supplementary Figure S1c).

The burden disproportionately affected older adults with aged ≥65 years. As illustrated in Supplementary Figure S2, temporal variations in the proportional distribution of ambient PM_2.5_-attributable IHD deaths and DALYs across age groups revealed persistent concentration in older adult populations. Throughout the study period, approximately 50% of global ambient PM_2.5_-attributable IHD deaths and DALYs consistently occurred among individuals aged 65 years or older.

#### Global burden of IHD by regions

In 2021, middle SDI regions exhibited the highest ASMR and ASDR for IHD attributable to ambient PM_2.5_, while high-SDI regions recorded the lowest values for both metrics. Between 1990 and 2021, high-SDI regions demonstrated the most substantial decline in ASMR, with an AAPC of −4.31 (95% CI: −4.5~−4.11). In contrast, low-middle-SDI regions experienced the largest ASMR increase (AAPC: 1.94; 95% CI: 1.47~2.41). A parallel pattern emerged for ASDR trends: high-SDI regions achieved the greatest reduction (AAPC: −3.98; 95% CI: −4.21~−3.74), whereas low-middle-SDI regions showed the steepest rise (AAPC: 1.91; 95% CI: 1.64~2.18). As shown in Table 1, various regions have distinct trends. For example, the ASMR in the South Asia region increased from 12.03 (95% UI: 76.55~19.16) in 1990 to 31.37 (95% UI: 18.8~43.77) in 2021 (AAPC: 3.04; 95%CI: 2.88~3.17), and the ASDR rose from 288.24 (95% UI: 156.91–462.21) to 703.17 (95% UI: 420.75~977.58) (Table 1).

### Association between ambient PM_2.5_-attributable IHD burden and SDI

**Figure 1:**
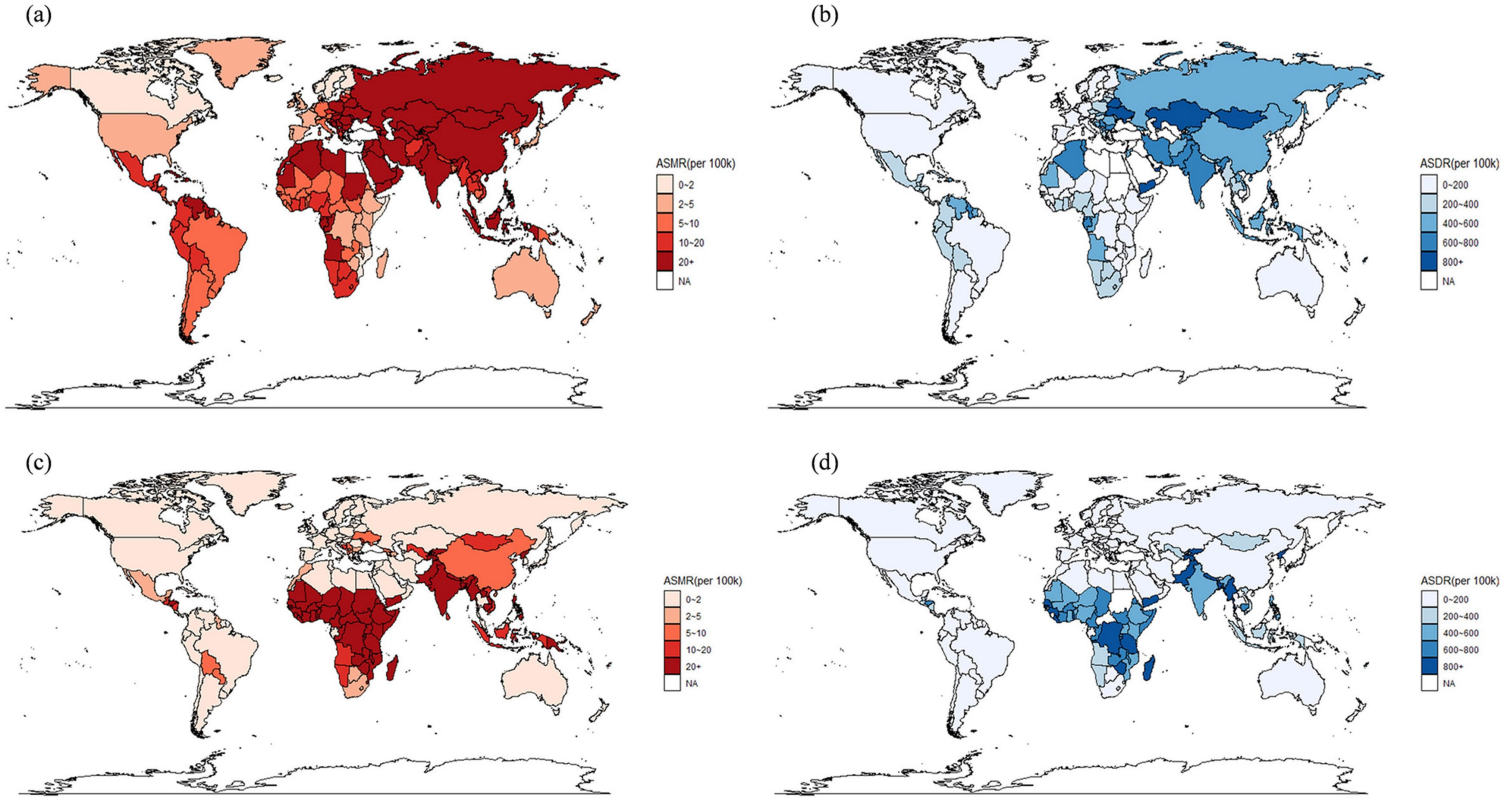
Global ASMR (per 100 k) **(a,c)** and ASDR (per 100 k) **(b,d)** for ischemic heart disease attributable to ambient PM_2.5_ **(a,b)** and household **(c,d)** in 2021.

Globally, substantial national disparities in ambient PM_2.5_-attributable IHD burden were observed in 2019, with over 20 times variations in ASMR across countries (Figure 1a). Asian countries and regions exhibited disproportionately high burdens compared to other regions. In contrast, Western Europe countries and regions, Northern Europe countries and regions, the America, Australia, and Eastern Africa countries and regions demonstrated relatively low ambient PM_2.5_-attributable IHD burdens (Figure 1).

**Figure 2:**
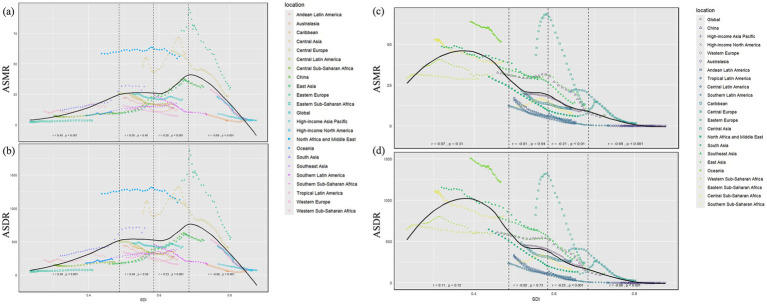
ASMR **(a,c)** and ASDR **(b,d)** for ischemic heart disease attributable to ambient PM_2.5_ **(a,b)** and household **(c,d)** across 21 geographical GBD regions by the SDI for both sexes combined from 1990 to 2021.

Visual correlation analysis between ambient PM_2.5_-attributable IHD burden [assessed via ASMR (Figure 2a) and ASDR (Figure 2b)], and SDI variations revealed distinct patterns when stratifying SDI into four intervals with cut-off points at approximately 0.459, 0.548, and 0.623. In SDI regions below 0.459, ambient PM_2.5_-attributable IHD burden showed a strong positive correlation with SDI progression. A weak positive association emerged in the 0.548~0.623 SDI range, while regions exceeding 0.623 SDI displayed a strong inverse correlation (Figure 2). For instance, in regions with SDI below 0.459, such as some Sub-Saharan African countries, the ASMR and ASDR values are relatively high and show an upward trend with increasing SDI. However, in regions with SDI above 0.623, like Western Europe and North America, the ASMR and ASDR values are low and decrease further as SDI increases (Figure 2).

**Figure 3:**
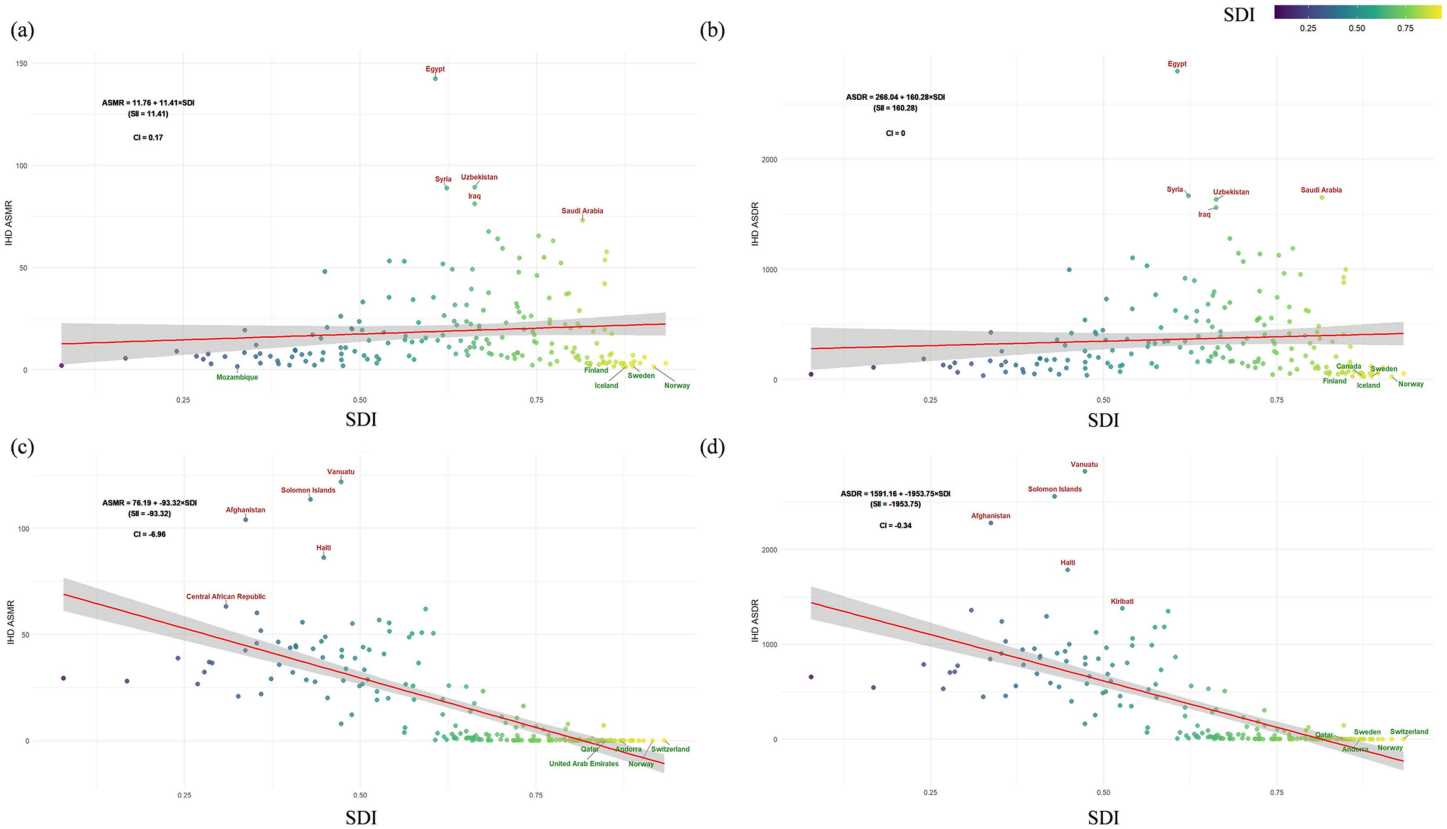
ASMR **(a,c)** and ASDR **(b,d)** for ischemic heart disease attributable to ambient PM_2.5_ **(a,b)** and household **(c,d)** by SDI across 204 countries, with inequality measures (SII/CI), both sexes in 2021.

**Figure 4:**
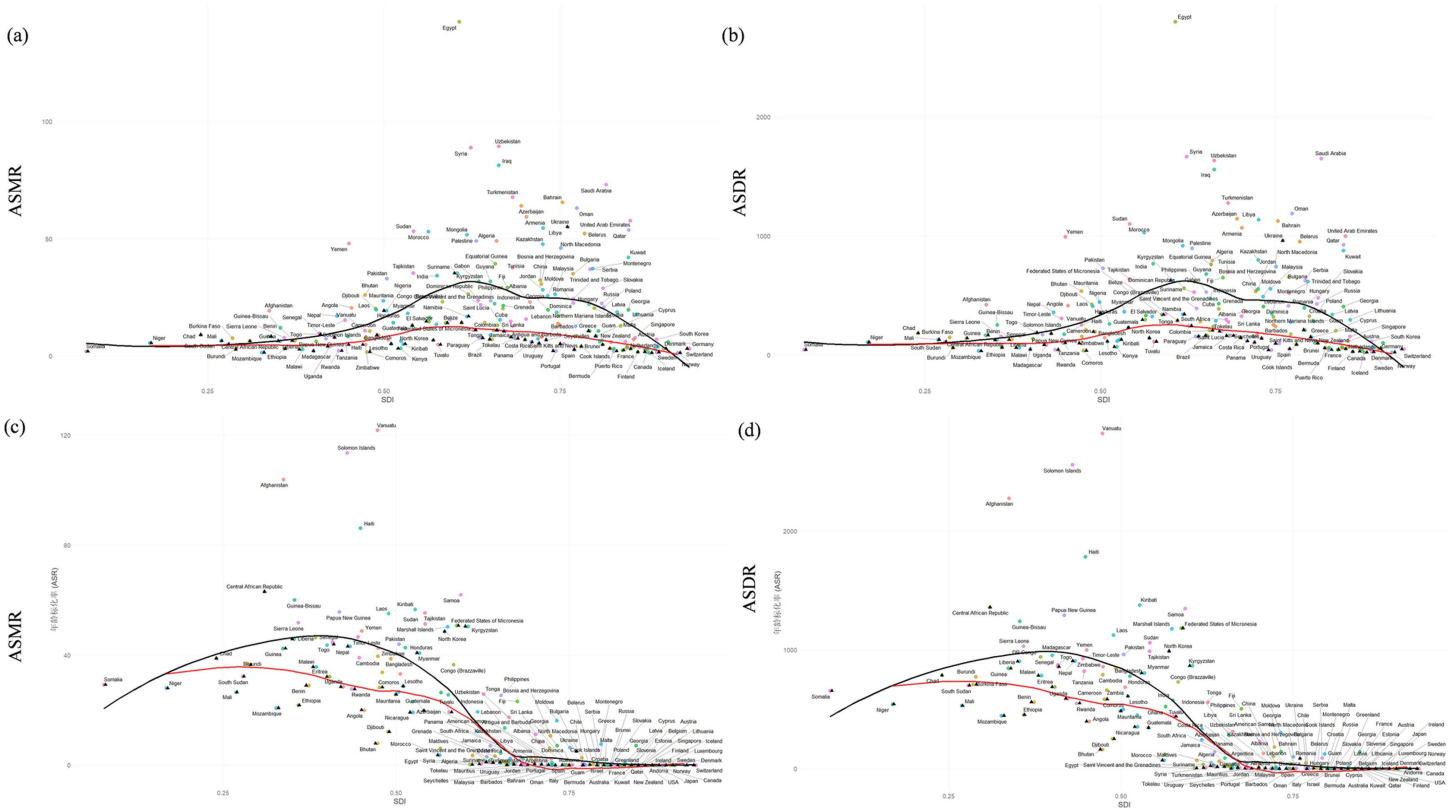
ASMR **(a,c)** and ASDR **(b,d)** for ischemic heart disease attributable to ambient PM_2.5_ **(a,b)** and household **(c,d)** across 204 GBD countries by the SDI for both sexes combined in 2021 (Black line) and frontier analysis (Red line).

Further analysis using the Slope Index of Inequality (SII) and Concentration Index (CI) revealed significant disparities in ambient PM2.5-attributable IHD burden among countries. For ASMR, the SII value of 11.41 indicates a substantial absolute disparity in IHD mortality rates across different SDI levels, with the CI at 0.17, suggesting a pro-rich distribution of the burden. Similarly, the ASDR analysis showed an SII of 160.28, highlighting significant absolute differences in disease burden, with a CI of 0.00, indicating no significant relative inequality. Figure 3 illustrates these inequalities, with countries like Egypt positioned above the expected trend line, indicating higher-than-expected IHD burden given their SDI, while countries such as Norway and Iceland lie below the trend line, demonstrating lower-than-expected burdens. These findings underscore the uneven distribution of IHD burden attributable to ambient PM_2.5_ exposure across countries with varying sociodemographic profiles (Figures 3a,b). The analysis of 204 countries revealed uneven distribution of IHD burden across different SDI levels. Countries in the low SDI group, such as those in Sub-Saharan Africa and South Asia, showed significantly higher ASMR and ASDR values (Figures 4a,b). Frontier analysis indicated that regions with higher SDI generally had lower IHD burdens. The red curve in Figure 4 represents the frontier of achievable reduction in IHD burden as SDI increases. Countries positioned above the frontier curve, such as Egypt, had higher IHD burdens than expected for their SDI level, while those below the curve, such as Spain, had lower burdens than expected.

### Decomposition of ambient PM_2.5_-attributable IHD burden

**Figure 5:**
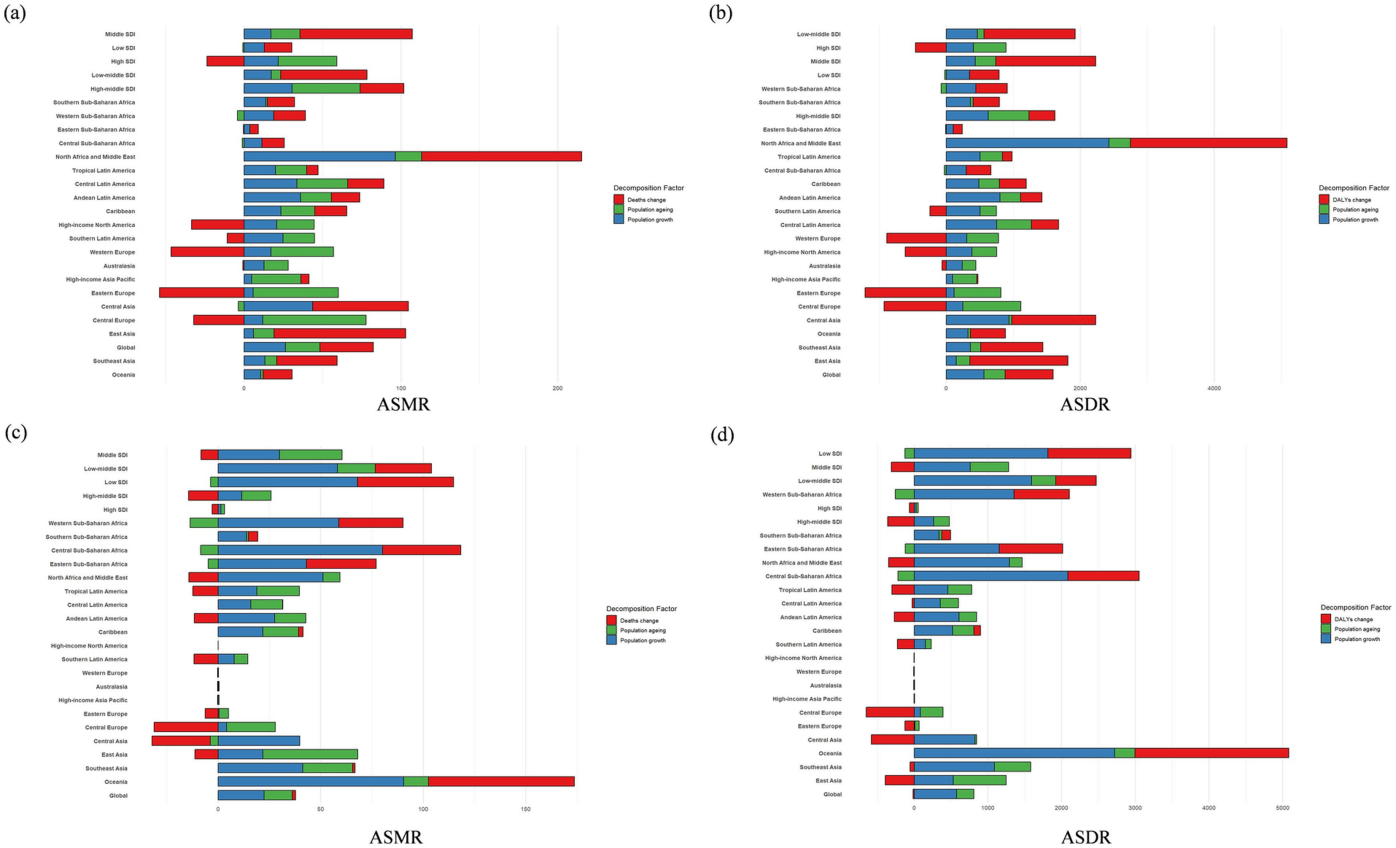
Decomposition of ASMR **(a,c)** and ASDR **(b,d)** for ischemic heart disease attributable to ambient PM_2.5_ **(a,b)** and household **(c,d)** across global, SDI, and GBD regions, both sexes in 2021.

Using a newly developed decomposition method, we analyzed the changes in the total number of IHD deaths and DALYs attributable to ambient PM_2.5_ across the five SDI groups and 21 GBD regions from 1990 to 2021 (Figures 5a,b). The decomposition reveals the contributions of population growth, population aging, and changes in mortality or DALY rates.

In middle SDI regions, changes in mortality rates were the dominant contributor to IHD burden. Low SDI regions showed a relatively even contribution from all three factors, reflecting ongoing demographic transitions and health challenges. High SDI regions experienced minimal contribution from mortality rate changes, with population growth and aging being the primary drivers of burden.

Among the GBD regions, North Africa and the Middle East had the highest burden, driven mainly by mortality rate changes. In contrast, Europe, the Americas, and Latin America saw declines in mortality rates, leading to a net decrease in IHD burden. However, population aging still contributed significantly to the burden in these regions. In Asia and Africa, despite some progress in reducing mortality rates, the substantial contributions from population growth resulted in a net increase in IHD burden.

### Forecast of ambient PM_2.5_-attributable IHD burden for the next 25 years

**Figure 6:**
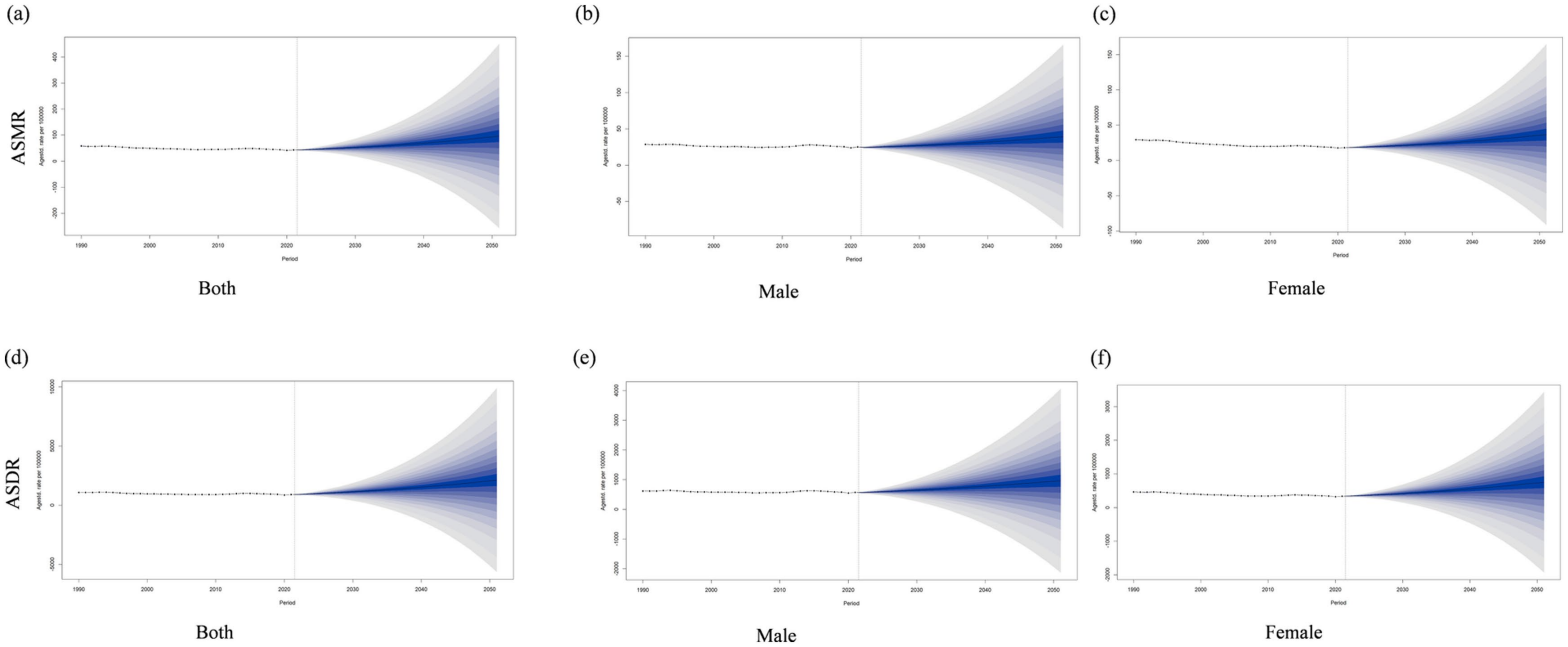
Projected trends for the next 25 years (2022–2046) in ambient PM_2.5_-attributable ischemic heart disease burden in Global: ASMR for both sexes **(a)**, males **(b)**, and females **(c)**; ASDR for both sexes **(d)**, males **(e)**, and females **(f)**.

Figure 6 presents the projected trends of ambient PM_2.5_-attributable IHD burden over the next 25 years (2022–2046) globally, analyzed by ASMR and ASDR, and stratified by gender. For ASMR (Figures 6a–c), the trends for both genders, males and females show a relatively stable pattern before 2022, followed by a growing divergence in the projected rates thereafter. This indicates an expected increase in the mortality impact of ambient PM_2.5_-attributable IHD in the coming decades, with the spread of the projection intervals suggesting uncertainties in the magnitude of this increase. Regarding ASDR (Figures 6d–f), similar to ASMR, a stable trend prior to 2022, and then an expanding range of estimated burden. The upward trend in the central estimates and the widening intervals imply that not only is the ASDR of ambient PM_2.5_-attributable IHD likely to rise, but there is also considerable variability in the potential extent of this burden across different scenarios.

### IHD burden attributable to household solid fuel PM_2.5_ from 1990 to 2021

#### Global burden of IHD

The global ASMR for IHD attributable to household solid fuel PM_2.5_ declined from 19.51 (95% UI: 14.16~26.53) per 100,000 in 1990 to 9.02 (95% UI: 4.85~16.3) per 100,000 in 2021, with a global average AAPC of −2.49 (95% CI: −2.64~−2.35) (Supplementary Figure S3a). Males showed a marginally smaller reduction (AAPC: −2.49; 95% CI: −2.69~−2.30) compared to females (AAPC: −2.55; 95% CI: −2.69~−2.41) (Supplementary Figure S3b). The ASDR similarly decreased from 456.4 (95% UI: 337.88~603.97) to 210.56 (95% UI: 118.9~365.27) per 100,000 over this period (Supplementary Figure S3c). The global AAPC for ASDR was −2.48 (95% CI: −2.60~−2.36) (Supplementary Figure S3d), with males exhibiting a slightly weaker decline (AAPC: −2.41; 95% CI: −2.58~−2.24) (Supplementary Figure S3e) than the global average, while females demonstrated a more pronounced reduction (AAPC: −2.59; 95% CI: −2.74~−2.44) (Supplementary Figure S3f). Mortality and DALY trends followed analogous trajectories (Table 2).

**Table 2:** ASMR, ASDR, and AAPCs attributed to Household PM_2.5_ from solid fuels in 1990 and 2021.

Location	Gender	ASMR (95%UI)			ASDR (95%UI)		
		1990	2021	1990–2021 AAPC	1990	2021	1990–2021 AAPC
Global	Both	19.51 (14.16, 26.53)	9.02 (4.85, 16.3)	−2.49^*^ (−2.64, −2.35)	456.4 (337.88, 603.97)	210.56 (118.9, 365.27)	−2.48^*^ (−2.6, −2.36)
	Male	22.98 (16.71, 30.78)	10.75 (5.68, 19.8)	−2.49^*^ (−2.69, −2.3)	544.48 (402.76, 720.28)	256.59 (141.03, 456.59)	−2.41^*^ (−2.58, −2.24)
	Female	16.73 (12.09, 23.11)	7.54 (4.03, 13.53)	−2.55^*^ (−2.69, −2.41)	377.1 (279.68, 499.7)	167.85 (95.07, 286.06)	−2.59^*^ (−2.74, −2.44)
SDI rank							
Low	Both	45.78 (35.86, 56.9)	40.63 (31.24, 50.09)	−0.34^*^ (−0.55, −0.14)	1017.01 (793.99, 1266.51)	853.61 (654.99, 1060.02)	−0.57^*^ (−0.69, −0.45)
	Male	48.35 (37.68, 61.14)	46.95 (35.28, 58.99)	−0.08 (−0.24, 0.06)	1114.54 (859.46, 1414.57)	1016.13 (757.18, 1287.85)	−0.25^*^ (−0.41, −0.1)
	Female	42.98 (32.73, 54.97)	34.78 (26.36, 44.23)	−0.70^*^ (−0.98, −0.43)	913.83 (700.28, 1163.13)	698 (530.58, 897.36)	−0.85^*^ (−0.98, −0.72)
Low-middle	Both	45.37 (34.95, 56.2)	28.85 (17.29, 42.01)	−1.43^*^ (−1.8, −1.08)	1050.75 (812.14, 1290.17)	634.58 (377.95, 928.71)	−1.60^*^ (−1.87, −1.35)
	Male	48.19 (37.48, 60.5)	33.41 (19.1, 50.11)	−1.15^*^ (−1.62, −0.69)	1165.47 (900.41, 1449.83)	760.27 (431.24, 1143.59)	−1.37^*^ (−1.68, −1.08)
	Female	42.32 (32.28, 52.98)	24.72 (14.99, 34.77)	−1.72^*^ (−2.17, −1.27)	929.1 (710.43, 1159.9)	516.07 (313.06, 723.29)	−1.92^*^ (−2.07, −1.77)
Middle	Both	29.54 (21.71, 38.06)	7.65 (1.77, 19.59)	−4.34^*^ (−4.77, −3.92)	612.28 (452.39, 783.09)	147.53 (35.14, 373.41)	−4.56^*^ (−4.86, −4.27)
	Male	31.81 (23.36, 41.77)	8.57 (1.75, 23.57)	−4.24^*^ (−4.83, −3.67)	690.08 (508.97, 899.31)	172.78 (37.83, 469.91)	−4.45^*^ (−4.76, −4.14)
	Female	27.24 (20.31, 35.57)	6.83 (1.72, 16.4)	−4.44^*^ (−4.88, −4)	533.05 (400.15, 691.68)	123.79 (32.3, 294.1)	−4.69^*^ (−5.05, −4.33)
High-middle	Both	14.34 (8.34, 26.6)	1.96 (0.14, 9.62)	−6.32^*^ (−6.99, −5.66)	293.3 (179.21, 513.25)	34.76 (2.5, 170.71)	−6.75^*^ (−7.41, −6.1)
	Male	16.85 (9.72, 30.77)	2.08 (0.12,10.29)	−6.63^*^ (−7.29,-5.98)	353.58 (210.43, 628.21)	38.72 (2.36, 189.83)	−7.00^*^ (−7.67, −6.34)
	Female	12.59 (7.29, 23.74)	1.85 (0.14, 8.76)	−6.15^*^ (−6.68, −5.62)	244.32 (148.09, 426.89)	30.94 (2.33, 145.93)	−6.53^*^ (−7.3, −5.77)
High	Both	1.56 (0.43, 4.07)	0.03 (0, 0.33)	−11.7^*^ (−12.2, −11.29)	32.99 (9.27, 84.91)	0.61 (0, 5.98)	−12.21^*^ (−12.74, −11.69)
	Male	1.81 (0.47, 4.91)	0.03 (0, 0.35)	−12.1^*^ (−12.53, −11.67)	40.93 (10.55, 110)	0.71 (0, 7.07)	−12.40^*^ (−12.86, −11.93)
	Female	1.35 (0.4, 3.39)	0.03 (0, 0.29)	−11.5^*^ (−12.18, −10.85)	25.67 (7.95, 62.99)	0.5 (0, 4.62)	−12.07^*^ (−12.84, −11.3)
GBD regions							
Andean Latin America	Both	17.13 (7.72, 27.04)	2.35 (0.38, 7.63)	−6.27^*^ (−6.48, −6.1)	337.14 (151.73, 537.17)	46.22 (7.64, 146.76)	−6.29^*^ (−6.51, −6.12)
	Male	17.17 (7.28, 28.73)	2.37 (0.36, 7.63)	−6.21^*^ (−6.55, −6.01)	361.39 (152.64, 602.16)	50.03 (7.96, 161.08)	−6.22^*^ (−6.47, −6.05)
	Female	16.98 (7.86, 25.78)	2.32 (0.41, 7.22)	−6.28^*^ (−6.56, −6.09)	312.55 (144.49, 481.54)	42.36 (7.6, 128.96)	−6.36^*^ (−6.61, −6.17)
Australasia	Both	0.15 (0, 1.76)	0 (0, 0.02)	−12.53^*^ (−12.82, −12.02)	2.76 (0, 31.41)	0.05 (0, 0.28)	−12.52^*^ (−12.78, −12.05)
	Male	0.17 (0, 1.92)	0 (0, 0.02)	−12.18^*^ (−12.45, −11.65)	3.28 (0, 37.24)	0.06 (0, 0.33)	−12.21^*^ (−12.5, −11.72)
	Female	0.14 (0, 1.6)	0 (0, 0.01)	−12.87^*^ (−13.2, −12.34)	2.23 (0, 25.53)	0.03 (0, 0.22)	−13.01^*^ (−13.3, −12.55)
Caribbean	Both	21.6 (13.78, 31.66)	10.48 (6.98, 15.09)	−2.31^*^ (−2.35, −2.27)	484.03 (317.92, 686.02)	264.7 (175.39, 381.57)	−1.94^*^ (−1.97, −1.9)
	Male	20.87 (12.76, 31.25)	10.79 (6.92, 15.89)	−2.05^*^ (−2.1, −2.01)	492.93 (310.44, 709.76)	277.59 (174.95, 405.18)	−1.81^*^ (−1.86, −1.76)
	Female	22.08 (14.46, 31.31)	10.19 (6.64, 15.22)	−2.47^*^ (−2.51, −2.43)	473.21 (316.9, 665.91)	252.51 (164.89, 386.3)	−2^*^ (−2.04, −1.97)
Central Asia	Both	43.49 (20.05, 77.92)	13.23 (5.19, 29.8)	−3.8^*^ (−3.94, −3.7)	822.29 (374.52, 1492.7)	242.54 (95.82, 542.07)	−3.92^*^ (−4.12, −3.8)
	Male	51.09 (22.71, 95.8)	14.94 (5.91, 34.21)	−3.93^*^ (−4.05, −3.83)	1019.24 (444.16, 1945.68)	283.18 (110.52, 643.94)	−4.08^*^ (−4.26, −3.94)
	Female	38.44 (17.87, 66.2)	12.05 (4.88, 27.22)	−3.71^*^ (−3.85, −3.62)	674.41 (314.52, 1158.42)	210.13 (86.1, 465.47)	−3.76^*^ (−3.93, −3.65)
Central Europe	Both	21.08 (5.01, 54.95)	1.64 (0.05, 10.75)	−8.01^*^ (−8.2, −7.9)	396.23 (96.01, 1038.52)	28.59 (0.89, 185.98)	−8.21^*^ (−8.42, −8.09)
	Male	24.53 (5.75, 67.65)	1.78 (0.05, 11.71)	−8.19^*^ (−8.4, −8.04)	501.2 (119.22, 1373.42)	34.04 (0.95, 226.59)	−8.39^*^ (−8.6, −8.22)
	Female	18.27 (4.3, 44.92)	1.49 (0.05, 9.72)	−7.87^*^ (−8.04, −7.76)	305.42 (74.36, 744.41)	23.4 (0.8, 150.08)	−8.05^*^ (−8.28, −7.95)
Central Latin America	Both	12.3 (5.3, 23.15)	3.67 (1.24, 9.21)	−3.99^*^ (−4.06, −3.92)	236.69 (102.24, 444.59)	70.09 (24.31, 176.49)	−4.03^*^ (−4.11, −3.96)
	Male	12.19 (4.96, 24.3)	3.93 (1.14, 10.41)	−3.74^*^ (−3.83, −3.66)	251.42 (102.11, 497.38)	79.13 (23.94, 210.26)	−3.74^*^ (−3.89, −3.66)
	Female	12.43 (5.56, 22.44)	3.43 (1.19, 8.54)	−4.21^*^ (−4.3, −4.13)	222.9 (101.1, 400.67)	62.05 (22.87, 148.89)	−4.21^*^ (−4.28, −4.15)
Central Sub-Saharan Africa	Both	53.08 (38.87, 71.64)	39.64 (26.9, 53.25)	−0.94^*^ (−1, −0.88)	1109.44 (804.07, 1508.81)	804.41 (539.65, 1081.76)	−1.03^*^ (−1.11, −0.95)
	Male	61.74 (42.17, 81.69)	47.89 (32.01, 65.77)	−0.81^*^ (−0.87, −0.75)	1370.08 (933.8, 1829.4)	1022.75 (672.78, 1417.14)	−0.95^*^ (−1.01, −0.9)
	Female	45.52 (30.04, 64.32)	33.08 (19.4, 47.81)	−1.02^*^ (−1.08, −0.96)	881.14 (577.52, 1276.39)	616.16 (364.96, 906.94)	−1.14^*^ (−1.21, −1.08)
East Asia	Both	32.24 (24.25, 41.43)	6.68 (1.46, 20.22)	−5.04^*^ (−5.37, −4.84)	604.68 (453.9, 777.52)	116.06 (27.39, 342.39)	−5.28^*^ (−5.61, −5.14)
	Male	35.53 (26.4, 47.62)	7.59 (1.48, 25.22)	−4.97^*^ (−5.32, −4.77)	664.24 (485.08, 889.17)	135.46 (30.11, 436.77)	−5.11^*^ (−5.52, −4.97)
	Female	30.2 (22.63, 38.85)	6.01 (1.46, 17.05)	−5.14^*^ (−5.53, −4.97)	555.08 (415.36, 714.55)	98.66 (24.97, 278.88)	−5.5^*^ (−5.8, −5.36)
Eastern Europe	Both	6.27 (1.38, 23.74)	1.65 (0.23, 6.52)	−4.27^*^ (−4.51, −4)	108.66 (24.13, 418.16)	28.72 (4, 115.28)	−4.37^*^ (−4.66, −4.05)
	Male	7.34 (1.62, 27.12)	1.75 (0.24, 7.24)	−4.6^*^ (−4.86, −4.32)	136.2 (29.67, 518.84)	33.74 (4.63, 141.03)	−4.54^*^ (−4.84, −4.22)
	Female	5.68 (1.27, 21.48)	1.54 (0.21, 6.08)	−4.19^*^ (−4.42, −3.92)	90.74 (21.19, 342.11)	24.42 (3.45, 96.17)	−4.16^*^ (−4.44, −3.87)
Eastern Sub-Saharan Africa	Both	29.24 (22.26, 36.31)	28.86 (22.14, 35.8)	−0.05^*^ (−0.08, −0.03)	648.87 (500.86, 807.97)	607.08 (461.12, 749.95)	−0.24^*^ (−0.27, −0.21)
	Male	31.99 (24.47, 40.82)	35.01 (26.49, 43.47)	0.29^*^ (0.26, 0.32)	749.17 (571.89, 945.32)	770.66 (576.63, 959.21)	0.08^*^ (0.05, 0.11)
	Female	26.41 (19.45, 34.49)	23.51 (17.29, 30.17)	−0.37^*^ (−0.41, −0.35)	548.12 (407.98, 722.15)	457.99 (347.45, 585.04)	−0.58^*^ (−0.6, −0.56)
High-income Asia Pacific	Both	0.12 (0.01, 0.79)	0 (0, 0.01)	−13.11^*^ (−13.24, −12.98)	2.25 (0.12, 14.5)	0.03 (0, 0.24)	−13.19^*^ (−13.31, −13.08)
	Male	0.11 (0.01, 0.79)	0 (0, 0.02)	−12.46^*^ (−12.59, −12.35)	2.5 (0.14, 16.13)	0.04 (0, 0.3)	−12.68^*^ (−12.8, −12.57)
	Female	0.11 (0.01, 0.78)	0 (0, 0.01)	−13.69^*^ (−13.83, −13.55)	1.95 (0.11, 12.67)	0.02 (0, 0.17)	−13.71^*^ (−13.94, −13.58)
High-income North America	Both	0.02 (0, 0.14)	0 (0, 0.01)	−8.38^*^ (−8.51, −8.24)	0.3 (0, 2.52)	0.02 (0, 0.12)	−8.15^*^ (−8.27, −8.01)
	Male	0.02 (0, 0.14)	0 (0, 0.01)	−8.22^*^ (−8.36, −8.04)	0.36 (0, 2.89)	0.03 (0, 0.14)	−8.16^*^ (−8.3, −7.97)
	Female	0.01 (0, 0.12)	0 (0, 0.01)	−8.45^*^ (−8.59, −8.33)	0.24 (0, 2.04)	0.02 (0, 0.1)	−8.26^*^ (−8.38, −8.13)
North Africa and Middle East	Both	30.07 (18.1, 47.96)	5.97 (3.8, 8.98)	−5.12^*^ (−5.16, −5.08)	644.54 (393.52, 1011.82)	132.38 (84.65, 196.76)	−5^*^ (−5.04, −4.97)
	Male	30.22 (18.09, 49.07)	6.15 (3.8, 9.49)	−5.03^*^ (−5.07, −5)	680.44 (408.8, 1106.36)	139.59 (87.33, 210.91)	−5.01^*^ (−5.04, −4.97)
	Female	29.67 (17.77, 47.29)	5.77 (3.58, 8.93)	−5.16^*^ (−5.2, −5.12)	604.23 (361.44, 941.41)	124.53 (78, 191.7)	−4.99^*^ (−5.03, −4.95)
Oceania	Both	63.85 (46.25, 85.24)	51.72 (37.06, 70.11)	−0.7^*^ (−0.74, −0.67)	1503.52 (1081.64, 2029.51)	1220.52 (855.1, 1664.86)	−0.69^*^ (−0.73, −0.66)
	Male	73.77 (51.32, 99.74)	60.18 (40.77, 84)	−0.7^*^ (−0.74, −0.68)	1824.72 (1251.72, 2497.37)	1486.01 (997.8, 2063.83)	−0.68^*^ (−0.73, −0.65)
	Female	53.41 (38.55, 73.02)	42.93 (30.54, 58.37)	−0.72^*^ (−0.76, −0.69)	1157.38 (831.45, 1603.93)	939.88 (659.42, 1274.53)	−0.69^*^ (−0.73, −0.66)
South Asia	Both	48.62 (37.5, 60.22)	28.59 (17.02, 43.78)	−1.69^*^ (−1.78, −1.61)	1156.8 (895.75, 1431.92)	639.9 (381.14, 976.68)	−1.88^*^ (−1.96, −1.82)
	Male	53.84 (40.73, 67.01)	34.24 (19.72, 54.29)	−1.42^*^ (−1.55, −1.31)	1321.64 (996.91, 1642.24)	778.54 (442.51, 1224.87)	−1.67^*^ (−1.75, −1.6)
	Female	42.84 (32.47, 53.45)	23.47 (13.99, 34.97)	−2^*^ (−2.16, −1.87)	973.04 (748.95, 1217.48)	506.53 (305.05, 748.59)	−2.14^*^ (−2.27, −2.05)
Southeast Asia	Both	36.2 (27.26, 46.21)	14.34 (6.47, 25.59)	−3^*^ (−3.13, −2.94)	792.23 (603.3, 1007.31)	301.29 (134.12, 541.94)	−3.13^*^ (−3.27, −3.08)
	Male	38.41 (27.75, 49.66)	15.39 (6.15, 29.06)	−2.95^*^ (−3.09, −2.9)	875.19 (637.72, 1127.64)	344.57 (137.89, 653.24)	−3.02^*^ (−3.17, −2.97)
	Female	34.07 (25.4, 43.59)	13.23 (6.44, 22.25)	−3.06^*^ (−3.19, −3.01)	714.54 (536.28, 912.78)	259.62 (125.28, 440.05)	−3.26^*^ (−3.39, −3.21)
Southern Latin America	Both	7.72 (1.9, 19.57)	0.19 (0, 1.74)	−11.34^*^ (−11.5, −11.23)	140.36 (34.75, 360.56)	3.68 (0.01, 34.11)	−11.15^*^ (−11.34, −11.02)
	Male	8.65 (1.97, 23.44)	0.22 (0, 2.12)	−11.25^*^ (−11.44, −11.12)	172.19 (38.23, 472.92)	4.73 (0.02, 45.23)	−11.01^*^ (−11.23, −10.88)
	Female	6.84 (1.79, 16.49)	0.16 (0, 1.41)	−11.54^*^ (−11.72, −11.42)	111.88 (29.52, 268.63)	2.74 (0.01, 24.45)	−11.42^*^ (−11.63, −11.29)
Southern Sub-Saharan Africa	Both	12.22 (7.31, 18.63)	7.62 (4.32, 13.18)	−1.49^*^ (−1.55, −1.42)	256.21 (151.61, 392.44)	162.71 (94.5, 276.11)	−1.48^*^ (−1.55, −1.41)
	Male	13.79 (7.78, 21.73)	7.81 (4.49, 14.14)	−1.81^*^ (−1.86, −1.75)	308.35 (171.69, 490.12)	182.5 (106.37, 330.12)	−1.69^*^ (−1.77, −1.63)
	Female	10.91 (6.74, 16.75)	7.24 (4.04, 12.09)	−1.31^*^ (−1.41, −1.2)	212.89 (133.13, 324.61)	144.39 (82.01, 242.85)	−1.29^*^ (−1.39, −1.19)
Tropical Latin America	Both	15.83 (8.29, 26.53)	1.45 (0.34, 3.86)	−7.45^*^ (−7.54, −7.39)	323.47 (168.14, 550.86)	32.04 (7.5, 85.31)	−7.24^*^ (−7.34, −7.17)
	Male	18.34 (9.53, 31.59)	1.7 (0.37, 4.65)	−7.51^*^ (−7.62, −7.42)	396.54 (203.88, 692.18)	39.21 (8.64, 108.07)	−7.24^*^ (−7.39, −7.16)
	Female	13.61 (7.12, 22.3)	1.24 (0.31, 3.13)	−7.45^*^ (−7.54, −7.39)	257.18 (134.88, 423.6)	25.85 (6.47, 64.34)	−7.15^*^ (−7.25, −7.09)
Western Europe	Both	0.11 (0, 0.96)	0 (0, 0.02)	−11.45^*^ (−11.55, −11.37)	2.12 (0.02, 17.64)	0.05 (0, 0.42)	−11.6^*^ (−11.71, −11.52)
	Male	0.13 (0, 1.13)	0 (0, 0.03)	−11.49^*^ (−11.61, −11.42)	2.71 (0.03, 22.77)	0.06 (0, 0.51)	−11.61^*^ (−11.72, −11.54)
	Female	0.1 (0, 0.79)	0 (0, 0.02)	−11.48^*^ (−11.61, −11.39)	1.61 (0.02, 12.86)	0.03 (0, 0.33)	−11.71^*^ (−11.83, −11.63)
Western Sub-Saharan Africa	Both	36.53 (27.02, 47.86)	31.15 (21.4, 42.19)	−0.51^*^ (−0.58, −0.46)	718.09 (532.4, 943.68)	600.15 (416.89, 816.79)	−0.58^*^ (−0.64, −0.52)
	Male	33.85 (22.72, 47.34)	31.6 (20.59, 44.38)	−0.19^*^ (−0.26, −0.14)	694.5 (467.67, 973.64)	636.38 (416.2, 900.9)	−0.25^*^ (−0.3, −0.2)
	Female	38.54 (27.57, 51.08)	30.73 (21.39, 42.45)	−0.72^*^ (−0.79, −0.65)	732.53 (525.81, 969.04)	567.46 (387.39, 790.84)	−0.81^*^ (−0.88, −0.74)

^*^Indicate that the AAPC is significantly different from zero at α = 0.05.

#### Global burden of IHD by gender and age

The ASMR for IHD attributable to household solid fuel PM_2.5_ was 10.75 (95% UI: 5.68~19.8) per 100,000 males and 7.54 (95% UI: 4.03~13.53) per 100,000 females. Similarly, males exhibited substantially higher ASDR compared to females (Table 2). Over the 31-year period, both genders demonstrated analogous trajectories in ASMR and ASDR reductions, though females experienced marginally greater declines than males (Supplementary Figure S3).

The burden disproportionately affected middle-aged and older populations. As shown in Supplementary Figure S4, temporal variations in the proportional distribution of household solid fuel PM_2.5_-attributable IHD deaths and DALYs across age groups revealed persistent concentration in older demographics. Approximately 50% of global deaths and DALYs attributable to household solid fuel PM_2.5_ exposure occurred among individuals aged ≥ 50 years during this period.

#### Global burden of IHD by regions

In 2021, low-SDI regions exhibited the highest ASMR and ASDR for IHD attributable to household solid fuel PM_2.5_, while high-SDI regions showed the lowest values for both metrics. Between 1990 and 2021, all SDI regions experienced declining trends in Household solid fuel PM_2.5-_attributable IHD burden, with high-SDI regions demonstrating the most substantial reductions: ASMR decreased at an AAPC of −11.7 (95% CI: −12.2~−11.29) and ASDR at −12.21 (95% CI: −12.74~−11.69). As shown in Table 2, various regions have distinct trends. For example, the ASMR in the Central Europe region decreased from 21.08 (95% UI: 5.01~54.95) in 1990 to 1.64 (95% UI: 0.05~10.75) in 2021 (AAPC: −8.01 95%CI: −8.2~−7.9), and the ASDR decreased from 396.23 (95% UI: 96.01~1038.52) to 28.59 (95% UI: 0.89~185.98), with an estimated change of −8.21 (95% UI: −8.42~−8.09) (AAPC: −8.21; 95%CI: −8.42~−8.09) (Table 2).

### Association between household solid fuel PM_2.5_-attributable IHD burden and SDI

Globally, substantial national disparities in household solid fuel PM_2.5-_attributable IHD burden were observed in 2021. African and South Asian nations exhibited disproportionately high burdens compared to other regions, while Europe, the Americas, and Australia demonstrated relatively low burdens (Figures 1c,d).

Loess smoothing curve analysis of Pearson correlation coefficients revealed distinct SDI-dependent patterns. In SDI regions below 0.459, household solid fuel PM_2.5_-attributable IHD burden showed a weak positive association with SDI progression. Conversely, regions exceeding an SDI of 0.623 demonstrated a strong inverse correlation between household solid fuel PM_2.5_-attributable burden and SDI levels (Figures 2c,d). To quantify health inequalities in IHD burden from household solid fuel PM_2.5_, we applied the Slope Index of Inequality (SII) and Concentration Index (CI). As presented in Figure 3 (ASMR in Figure 3a, ASDR in Figure 3b) for 204 countries over 1990–2021. Both ASMR (SII = −93.32, CI = −6.96) and ASDR (SII = −1953.75, CI = −0.34) showed negative SII and CI values, indicating that lower-SDI countries bear a disproportionately higher Household Solid Fuel PM_2.5_-attributable IHD burden in terms of both mortality and disability-adjusted life-years (Figures 3c,d). As ASMR and ASDR shown in Figure 4, 204 countries revealed uneven distribution of IHD burden across different SDI levels. Lower-SDI countries showed significantly higher ASMR/ASDR (clustered at higher y-values), while high-SDI regions exhibited minimal burdens (near the x-axis). This confirms low-SDI regions face disproportionate IHD burden from household solid fuel PM_₂.₅_. Frontier analysis of Figures 4c,d, low-SDI regions have a large gap between actual burdens and the frontier. High-SDI countries show smaller gaps (burdens approaching the frontier). Thus, SDI advancement benefits all regions, but low-SDI areas have the greatest reduction potential.

### Decomposition of household solid fuel PM_2.5_-attributable IHD burden

The decomposition of ischemic heart disease (IHD) burden attributable to household solid fuel PM_2.5_ exposures in 2021 was shown in Figures 5c,d. For ASMR, in low SDI regions, population growth was the primary driver of ASMR increase, followed by mortality change, with minimal contribution from population aging. In other regions (including low-middle, middle, high-middle, and high SDI areas, as well as West Africa, East Africa, North Africa, and the Middle East), ASMR changes were predominantly driven by mortality change and population growth, with negligible impact from population aging. Similarly, ASDR showed a consistent pattern.

In summary, population growth was the key driver of increased IHD burden in low SDI regions. In other regions, ASR changes and population growth jointly contributed to the burden, while population aging had minimal impact across all regions.

### Forecast of household solid fuel PM_2.5_-attributable IHD burden for the next 25 years

The projected trends over the next 25 years (2022–2046) in household solid fuel PM_2.5_-attributable IHD burden globally were shown in Figure S5. For both ASMR and ASDR, a declining trend is observed before the projection period. During the projection from 2022 to 2046, the uncertainty intervals (represented by the shaded areas) gradually expand. The predicted values suggest that the burden of IHD attributable to household solid fuel PM_₂.₅_ will tend to stabilize at a certain level for all groups (both genders, male, and female) by the end of the projection period, although there are differences in the magnitude of the burden among different gender.

## Discussion

This study elucidates the significant impacts of gender, age, and SDI levels on IHD burden through a comprehensive analysis of global patterns in IHD attributable to ambient PM_2.5_ and household solid fuel-derived PM_2.5_ exposure. Key findings indicate that ambient PM_2.5_ exposure demonstrates more pronounced effects on IHD burden among males and individuals aged ≥65 years, with the highest rates observed in middle SDI regions. Notably, South Asia emerged as the most severely affected area in 2021. Although age-standardized rates have declined slightly in high SDI countries, population growth and aging have offset much of the progress. In contrast, the burden of IHD attributable to household PM_₂.₅_ exposure has decreased substantially over time, particularly in low and low-middle SDI regions, reflecting reduced reliance on solid fuels. Nonetheless, a residual burden remains in low-SDI countries, indicating ongoing inequalities. These patterns align with recent epidemiological evidence (18), highlighting diverging health impacts from different PM_2.5_ emission sources.

Emerging evidence suggests that the toxicological profiles of ambient PM_2.5_ and household solid fuel-derived PM_2.5_ may differ substantially due to distinct sources, combustion processes and chemical compositions (19). Ambient PM_2.5_ in urban environments typically contains higher proportions of transition metals and black carbon from fossil fuel combustion (3), and has been strongly linked to ischemic heart disease (IHD) burden, particularly in regions with high population density and aging demographics. In contrast, household solid fuel-derived PM_2.5_, which enriched in polycyclic aromatic hydrocarbons and organic carbon (20), has shown a declining contribution to IHD burden over the past three decades, particularly in low and low-middle SDI countries. These compositional differences may explain the observed disparity in cardiovascular toxicity, with ambient PM_2.5_ demonstrating stronger associations with atherosclerotic progression compared to household solid fuel-derived PM_2.5_ (21). This pathophysiological distinction underscores the need for source-specific risk assessments in environmental health policies.

The health impacts of ambient PM_2.5_ on IHD burden exhibited marked disparities across SDI regions. In high-SDI regions, implementation of clean fuel adoption, advanced pollution control technologies, and robust healthcare resources has significantly mitigated IHD burden (18). Recent technological advancements in particle filtration systems have enabled high-income countries to achieve PM_2.5_ reductions exceeding 50% in urban centers since 2010 (22), in high-income countries, long-standing implementation of clean energy policies, advanced air quality regulations, and comprehensive healthcare infrastructure has led to a significant decline in IHD burden attributable to ambient PM₂.₅. Conversely, low-middle SDI regions face escalating IHD burdens linked to industrial expansion and population growth, which amplify PM_2.5_ exposure (23–25). Notably, ambient PM_2.5_ demonstrated divergent correlations with IHD burden across SDI strata (26): a strong positive association in low-SDI regions versus a strong inverse correlation in high-SDI regions. This dual socioeconomic dynamic reflects both the protective effects of pollution control technologies in developed economies and the exacerbating role of industrialization-driven PM_2.5_ exposure in transitioning regions.

The temporal dimension of PM_2.5_ exposure warrants particular attention. Longitudinal studies reveal that cumulative exposure over 10–15 years significantly elevates coronary calcium scores independent of current exposure levels (27). This latency effect suggests that current IHD burdens in developing regions may reflect historical pollution levels, while present control measures may require decades to manifest cardiovascular benefits-a critical consideration for policy evaluation timelines.

Household solid fuel-derived PM_2.5_ disproportionately affects low-SDI regions, attributable to widespread reliance on solid fuels for cooking and heating (28, 29). Innovative intervention studies demonstrate that advanced combustion stoves can reduce indoor PM_2.5_ concentrations by 60–80% while maintaining cultural cooking practices (30). Consistent with our findings, epidemiological evidence confirms declining household solid fuel use and associated IHD burden as socioeconomic development progresses (31). The strong inverse correlation observed in high-SDI regions underscores the cardiovascular benefits of clean energy transitions (32).

Gender and age disparities in PM_2.5_ susceptibility may stem from occupational exposures, comorbidities, and immunosenescence (33). Emerging evidence also implicates androgen-mediated enhancement of pulmonary oxidative stress pathways as a potential contributor to male vulnerability (34). Higher minute ventilation rates in males potentiate respiratory deposition and systemic translocation of PM_2.5_. Older adults exhibit heightened vulnerability due to age-related cardiopulmonary compromise and diminished PM_2.5_ detoxification capacity (35), necessitating targeted prevention strategies for these demographics (36).

Importantly, our SII and CI analysis demonstrated significant socioeconomic inequalities in IHD burden: ambient PM_₂.₅_ exhibited a positive SII (≈11.41) and CI (≈0.17), indicating a ‘pro-rich’ distribution, while household PM₂.₅ showed negative SII (≈ −93.3) and CI (≈ −6.96), reinforcing a ‘pro-poor’ burden concentration. These findings echo studies in China showing unequal PM_₂.₅_ exposure and health impact distributions across SES groups (37, 38). Furthermore, frontier analysis identified that many low- to middle-SDI countries-especially in South Asia and Sub-Saharan Africa—lag substantially behind the theoretical ‘health frontier’ for IHD burden given their SDI. This aligns with global inequality trends in PM_2.5_ exposures, where a small subset of countries bears disproportionate burden (39). These gaps highlight missed opportunities for efficient air quality control and cardiovascular health investment independent of economic growth. Decomposition analysis clarified that declining IHD burden in high-SDI regions is largely attributable to reduced exposure and improved healthcare, whereas in low-middle SDI regions, population aging and rising ambient PM_2.5_ exposure are the main drivers of increasing burden. This supports global findings that demographic transition and exposure escalation are key contributors to IHD mortality increases (40).

These findings inform evidence-based policymaking for IHD prevention. Low-middle SDI regions require prioritized air quality governance, including accelerated clean fuel adoption and emission reduction initiatives. In Mexico, meeting the WHO PM_2.5_ standard could prevent 3,600 premature deaths yearly, saving $3.8 billion (41). In Beijing, lowering PM_2.5_ to the national standard would cut medical costs and boost QALYs (35, 42). Concurrently, gender- and age-specific interventions, such as enhanced hypertension control and cardiovascular risk stratification-should be integrated into public health frameworks (43).

While leveraging GBD estimates and satellite-based PM_2.5_ assessments, potential exposure misclassification persists due to spatial–temporal resolution constraints. The analysis did not account for PM_2.5_ compositional heterogeneity, which may differentially influence cardiovascular toxicity (44). Future investigations should prioritize prospective cohorts in high-risk populations and elucidate source-specific PM_2.5_ component effects.

## Conclusion

Despite a global decline in PM_2.5_-attributable IHD burden from 1990 to 2021, divergent trends persisted across SDI strata. The epidemiological epicenter has shifted from high-SDI nations to low-middle-SDI regions, disproportionately impacting males and older adults. This transition reflects multifactorial drivers: rising PM_2.5_ exposure with inadequate mitigation, alongside aging populations and demographic growth in low-middle-SDI areas. Declining cardiovascular resilience and immunosenescence in aging populations amplify disease susceptibility under sustained PM_2.5_ exposure. Strengthened air quality governance to reduce ambient/household PM_2.5_ remains critical for alleviating IHD burden (45). Targeted pollution control optimizes healthcare resource allocation and stabilizes public health systems, particularly in regions grappling with environmental degradation and population aging.

## Data Availability

The original contributions presented in the study are included in the article/Supplementary material, further inquiries can be directed to the corresponding authors.

## Footnotes

^1^ https://ghdx.healthdata.org/gbd-2021/

^2^ https://www.dhsprogram.com/Data/

^3^ https://www.worldbank.org/en/programs/lsms/initiatives

^4^ https://cran.r-project.org
