# Supplementary material for: Global burden of ischemic heart disease attributable to ambient and household PM2.5 exposure: a comprehensive analysis (1990–2021) from socioeconomics perspective
Source: Front Public Health. 2025 Jul 10;13:1607163. doi: 10.3389/fpubh.2025.1607163 (PMC12287097; doi:10.3389/fpubh.2025.1607163)

Supplementary materials:

Figure S1 Temporal trends (1990-2021) in ambient PM2.5-attributable ischemic heart disease burden across regions: ASMR for both sexes (a), males (b), and females (c), ASDR for both sexes (d), males (e), and females (f).

Figure S2 Temporal trends (1990-2021) in the relative proportion of ambient PM2.5 related-ischemic heart disease burden: ASMR for both sexes (a), males (b), and females (c), ASDR for both sexes (d), males (e), and females (f).

Figure S3. Temporal trends (1990–2021) in household PM2.5 from solid fuels-attributable ischemic heart disease burden across regions: ASMR for both sexes (a), males (b), and females (c); ASDR for both sexes (d), males (e), and females (f).

Figure S4. Temporal trends (1990-2021) in the relative proportion of household PM2.5 from solid fuels related- ischemic heart disease burden: ASMR for both sexes (a), males (b), and females (c), ASDR for both sexes (d), males (e), and females (f).

Figure S5. Projected trends for the next 25 years (2022–2046) in household solid fuel PM2.5-attributable ischemic heart disease burden in Global: ASMR for both sexes (a), males (b), and females (c); ASDR for both sexes (d), males (e), and females (f).

Figure S1 Temporal trends (1990-2021) in ambient PM2.5-attributable ischemic heart disease burden across regions: ASMR for both sexes (a), males (b), and females (c), ASDR for both sexes (d), males (e), and females (f).

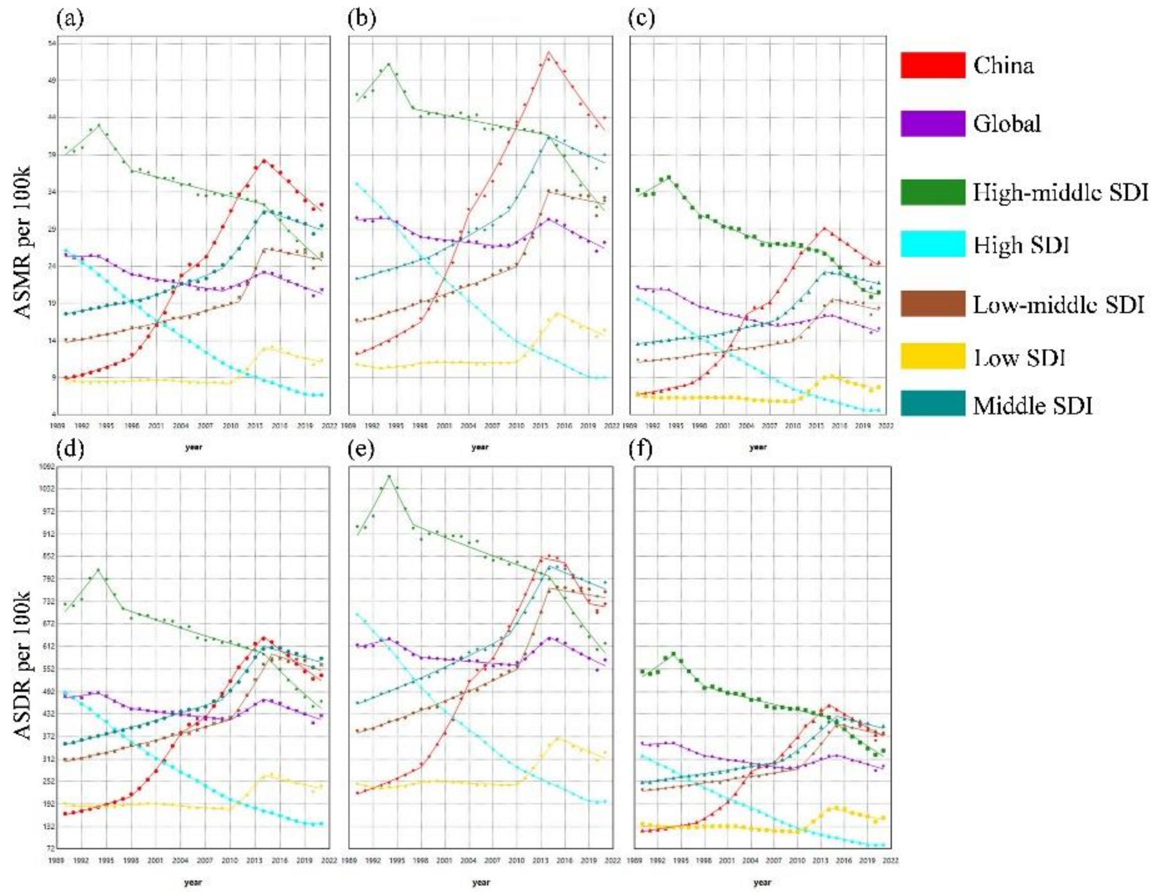

Figure S2 Temporal trends (1990-2021) in the relative proportion of ambient PM2.5 related-ischemic heart disease burden: ASMR for both sexes (a), males (b), and females (c), ASDR for both sexes (d), males (e), and females (f).

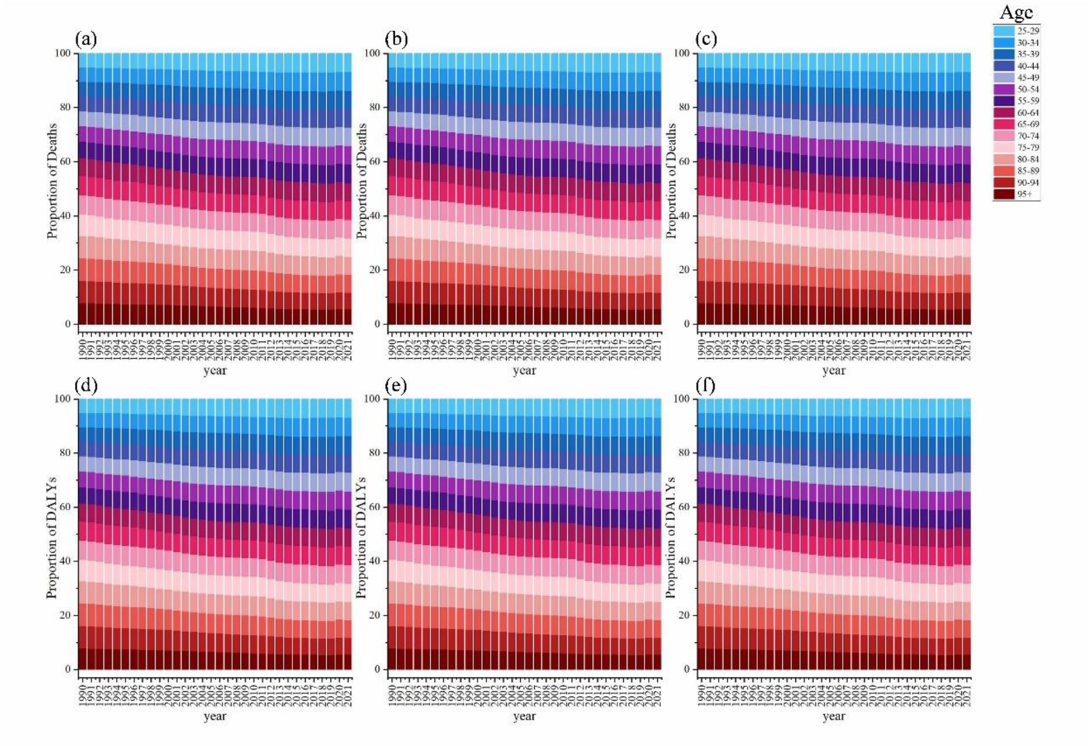

Figure S3. Temporal trends (1990–2021) in household PM2.5 from solid fuels-attributable ischemic heart disease burden across regions: ASMR for both sexes (a), males (b), and females (c); ASDR for both sexes (d), males (e), and females (f).

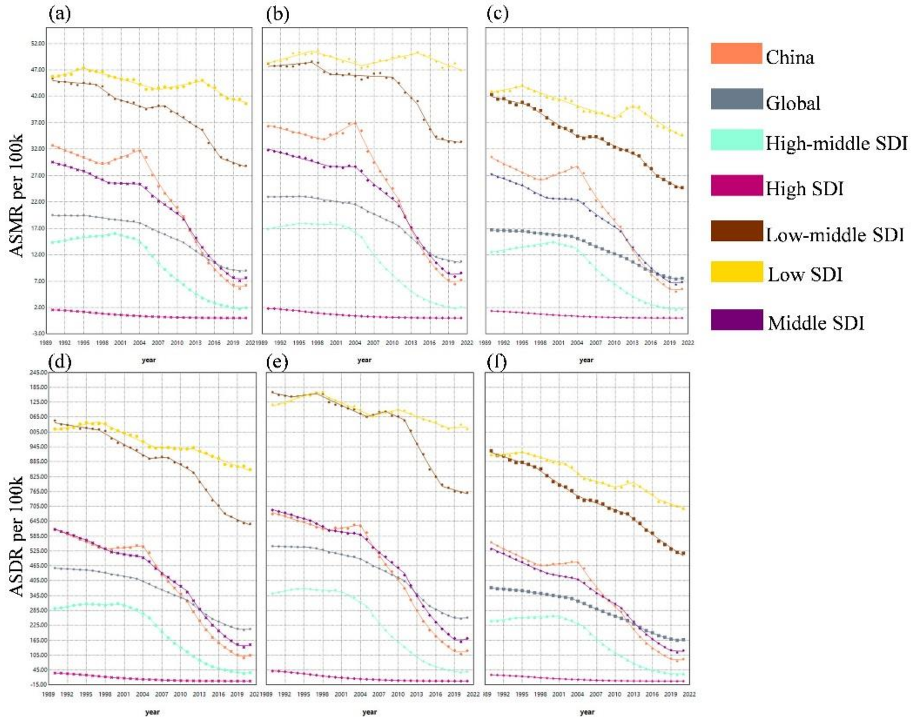

Figure S4. Temporal trends (1990-2021) in the relative proportion of household PM2.5 from solid fuels related- ischemic heart disease burden: ASMR for both sexes (a), males (b), and females (c), ASDR for both sexes (d), males (e), and females (f).

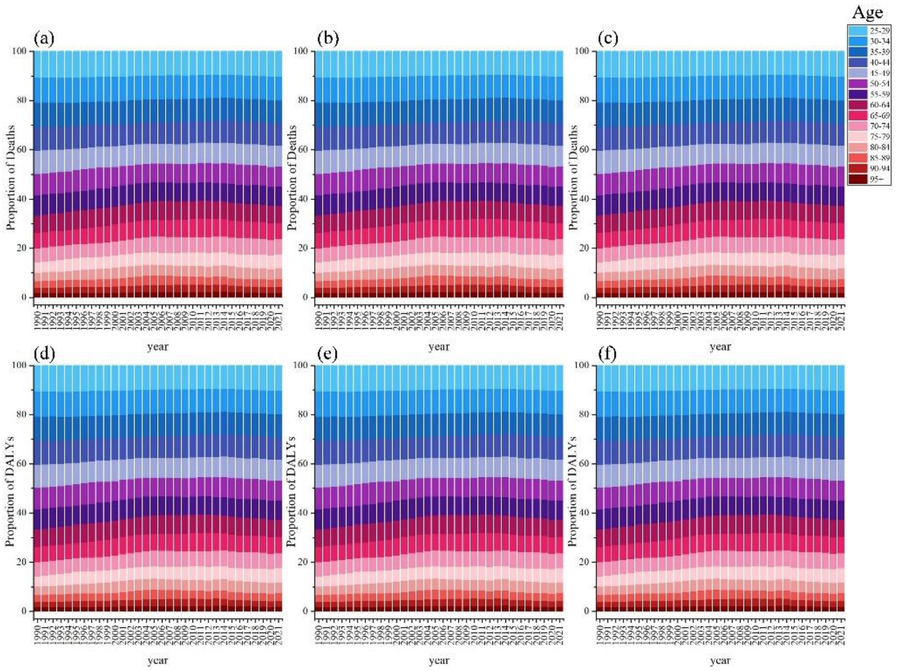

Figure S5. Projected trends for the next 25 years (2022–2046) in household solid fuel PM2.5-attributable ischemic heart disease burden in Global: ASMR for both sexes (a), males (b), and females (c); ASDR for both sexes (d), males (e), and females (f).

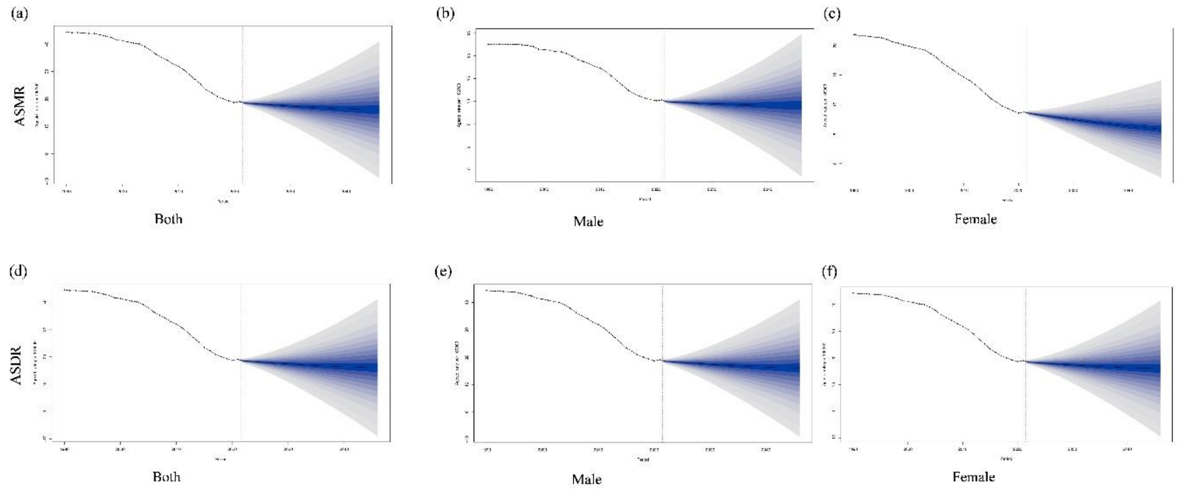

Supplement: Supplementary file 1 [file Data_Sheet_1.pdf]
